# Nanomaterial-Based Immunocapture Platforms for the Recognition, Isolation, and Detection of Circulating Tumor Cells

**DOI:** 10.3389/fbioe.2022.850241

**Published:** 2022-03-14

**Authors:** Yichao Liu, Rui Li, Lingling Zhang, Shishang Guo

**Affiliations:** ^1^ Center for Evidence-Based and Translational Medicine, Zhongnan Hospital of Wuhan University, Wuhan, China; ^2^ Xinjiang Key Laboratory of Solid State Physics and Devices, Xinjiang University, Urumqi, China.; ^3^ Key Laboratory of Artificial Micro- and Nano-structures of Ministry of Education, School of Physics and Technology, Wuhan University, Wuhan, China

**Keywords:** biological detection, circulating tumor cells, nanomaterials, immunocapture platform, liquid biopsy

## Abstract

Circulating tumor cells (CTCs) are a type of cancer cells that circulate in the peripheral blood after breaking away from solid tumors and are essential for the establishment of distant metastasis. Up to 90% of cancer-related deaths are caused by metastatic cancer. As a new type of liquid biopsy, detecting and analyzing CTCs will provide insightful information for cancer diagnosis, especially the in-time disease status, which would avoid some flaws and limitations of invasive tissue biopsy. However, due to the extremely low levels of CTCs among a large number of hematologic cells, choosing immunocapture platforms for CTC detection and isolation will achieve good performance with high purity, selectivity, and viability. These properties are directly associated with precise downstream analysis of CTC profiling. Recently, inspired by the nanoscale interactions of cells in the tissue microenvironment, platforms based on nanomaterials have been widely explored to efficiently enrich and sensitively detect CTCs. In this review, various immunocapture platforms based on different nanomaterials for efficient isolation and sensitive detection of CTCs are outlined and discussed. First, the design principles of immunoaffinity nanomaterials are introduced in detail. Second, the immunocapture and release of platforms based on nanomaterials ranging from nanoparticles, nanostructured substrates, and immunoaffinity microfluidic chips are summarized. Third, recent advances in single-cell release and analysis of CTCs are introduced. Finally, some perspectives and challenges are provided in future trends of CTC studies.

## 1 Introduction

The International Agency for Research on Cancer provided the cancer incidence and mortality in 2020, based on Global Cancer Statistics 2020 (Sung et al., 2021). A total of 185 countries and 36 kinds of cancers were analyzed, and the data demonstrated that female breast cancer has surpassed lung cancer as the most commonly diagnosed cancer, with an estimated 2.26 million new cases (11.7%), followed by lung cancer with 2.20 million new cases (11.4%), colorectal cancer with 1.93 million new cases (10.0%), prostate cancer with 1.41 million new cases (7.3%), and stomach cancer with 1.08 million new cases (5.6%). However, lung cancer remains the leading cause of cancer death due to its highly metastatic capacity, which has led to an estimated 1.8 million deaths in lung cancer patients. Although some of these solid tumors can be removed through surgery, for malignant tumors that grow in the epithelial tissue, these cancer cells grow rapidly, and invasion usually occurs in their surrounding tissues, which may lead to serious metastasis (Husemann et al., 2008; Sosa et al., 2014).

In 1869, Thomas Ashworth reported tumor-like cells that were found in the blood of a patient after death by metastatic cancer (Ashworth, 1869). Then, the concept of circulating tumor cells (CTCs) was proposed, these cells refer to all kinds of tumor cells that shed from cancerous tumors and enter the peripheral blood system, and they may have the ability to develop at other tumor sites and have important relationships with metastasis (Chaffer and Weinberg, 2011; Alix-Panabières and Pantel, 2013). Thus, CTCs have a substantial possibility of reflecting the genetic information of the primary tumor, such as genomic alterations (Ni et al., 2013; Lohr et al., 2014), gene expression (Yu et al., 2012; Kalinich et al., 2017), and protein expression (Kalinsky et al., 2015; Wallwiener et al., 2015). However, due to the extremely low concentration of CTCs among a large number of hematologic cells in the peripheral blood, the isolation of CTCs from clinical blood samples must be the first step for their further characterization and analysis.

Currently, numerous approaches have been reported to isolate CTCs from large amount of background blood cells, such as red blood cells (RBCs) and white blood cells (WBCs), due to their different physical and/or biological properties. Because tumor cells are usually larger than RBCs, and the high nucleo-cytoplasmic ratio (N/C ratio) of tumor cells causes the overall biomechanical properties of cancerous cells to differ from those of WBCs, CTC isolations based on the size (Hayata et al., 2010; Huang et al., 2014), deformability (Beech et al., 2012), density (Kim et al., 2014), or dielectric properties (Shim et al., 2013) of different cells have been established. These are usually label-free methods depending on the physical properties, and high-throughput cell isolation can be easily achieved. For example, a micropore-based membrane filter could process 7.5 ml of blood samples within 2 min (Lin et al., 2010), but the low purity of the obtained CTCs is a challenge for further analysis because CTCs have overlapping sizes with WBCs. Furthermore, the micropores of the filter clog when a large number of cells are processed, which may affect filtration and consequently squeeze cells. Hydrodynamic chromatography can achieve more rapid and higher throughput separation than filtration by using interactions between particles and obstacles in flow, which will result in different flow velocities based on the different sizes and deformability capacities of the cells (Beech et al., 2012). Unfortunately, leukocytes are difficult to separate from cancer cells because both cells are nucleated cells with similar deformability and size. Based on the different densities of various cells, Ficoll density gradient centrifugation (Gertler et al., 2003) was developed to separate mononuclear cells (including cancerous cells) from other blood cells. Centrifugation is a common pretreatment of blood in the clinic, but WBCs are also mononuclear cells, which may seriously lower the purity of isolated CTCs and affect the sensitivity of CTCs in further characterization. However, different mononuclear cells have different dielectric properties, and dielectrophoresis (DEP) (Shim et al., 2013) can be used to sort and enrich CTCs from the layer of mononuclear cells after centrifugation and is another method of label-free and low-cost CTC isolation. Moreover, the DEP method can be further improved by combining other separation techniques, such as an optically induced DEP system (Huang et al., 2013), but the performance of DEP in throughput is undesirable.

In addition to physical isolation methods, immunoaffinity methods based on the biological properties of CTCs present more specific and sensitive abilities in distinguishing CTCs, which can highly enhance the capture yield and purity of target cells. Immunocapture methods are usually based on the special expression of proteins or genes that are not expressed in other blood components. For example, some specific epithelial markers, cytokeratins (cytoskeletal proteins), and epithelial cell adhesion molecule (EpCAM) have been demonstrated to be expressed on the cytomembrane of CTCs (epithelial cells). Thus, immunoaffinity methods can accurately distinguish between CTCs and numerous background hematologic cells (Talasaz et al., 2009; Earhart et al., 2014). In 2007, Nagrath et al. developed a “CTC-chip” by using anti-EpCAM antibody–coated microposts in a microfluidic chip (Nagrath et al., 2007). This immunoaffinity platform achieved efficient and selective separation of viable CTCs from the peripheral blood samples, with an identified yield of CTCs reaching 99%, and achieved an approximately 50% purity. Currently, with the rapid development of nanotechnologies and nanomaterials, nanoscale-based immunocapture platforms have been established to further enhance the isolated yield. In short, nanomaterial-based immunocapture platforms for CTC detection with high sensitivity, high purity, and quick characterization will facilitate the advancement of cancer diagnosis and even personalized medical care.

This review article summarizes various immunocapture platforms based on different nanomaterials for the efficient isolation and sensitive detection of CTCs. The article starts from the design principles of immunoaffinity nanomaterials for capturing CTCs. Then, the immunocapture and release of platforms based on nanomaterials ranging from nanoparticles to nanostructured substrates and immunoaffinity microfluidic chips are summarized, together with their advantages and disadvantages. Recently, studies have shown that CTCs from a given patient may possess heterogeneous subpopulations, all of which may be related to the development of cancer metastasis (Keller and Pantel, 2019). Therefore, recent advances in single-cell release and analysis of CTCs are also introduced to further understand the inherent heterogeneity in CTCs. At the end of the review, we discuss some challenges of these nanomaterial-based immunocapture platforms that remain in clinical transformation.

## 2 Design Principles of Immunoaffinity Nanomaterials

**FIGURE 1:**
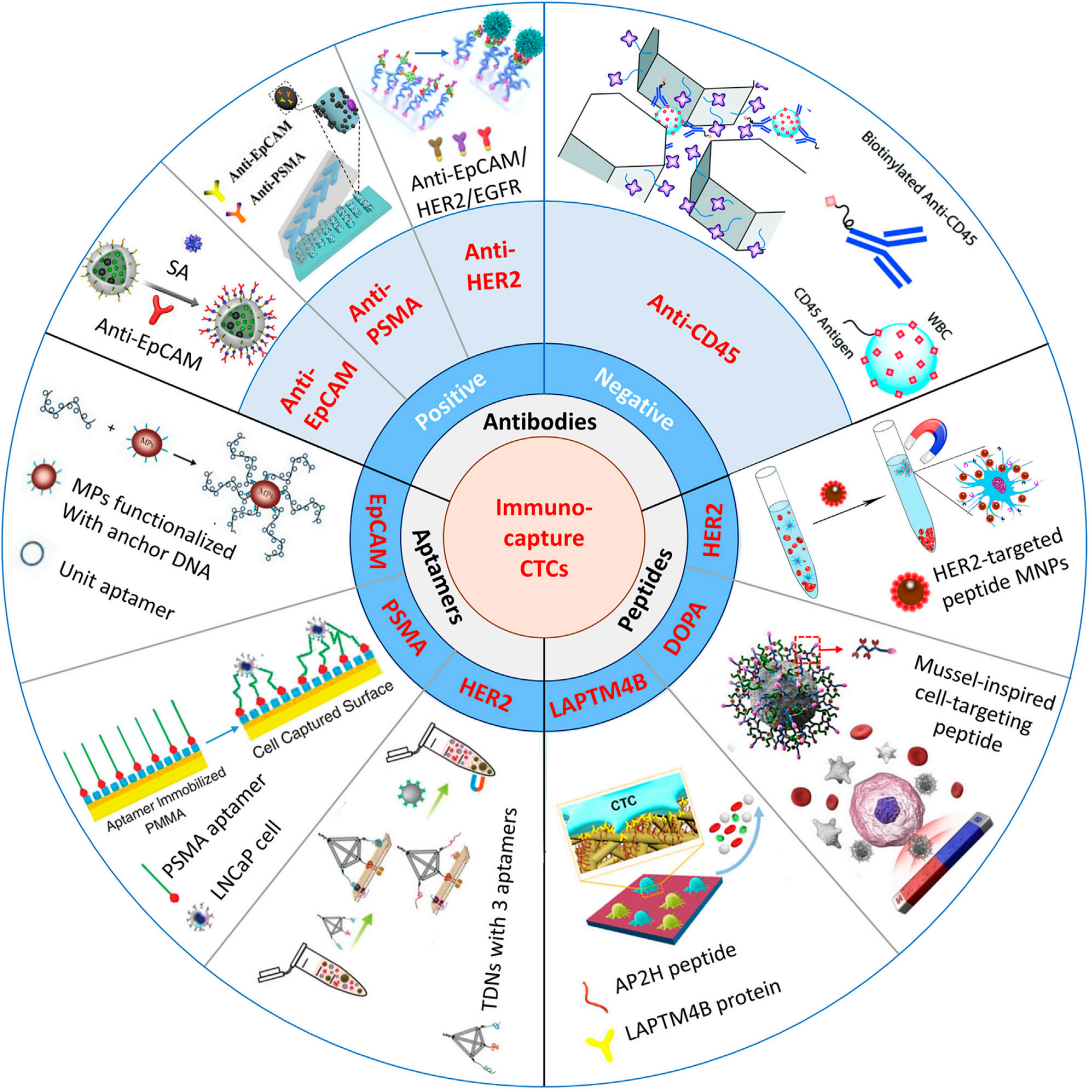
Schematic illustration of immunocapture platforms based on antibodies, peptides, and aptamers for CTC isolation. Positive antibodies: reproduced with permission from Cui et al. (2019), Copyright 2019, Elsevier. SA, streptavidin: reproduced with permission from Yin et al. (2018), Copyright 2018, American Chemical Society. PSMA, prostate-specific membrane antigen: reproduced with permission from Chen et al. (2019), Copyright 2019, John Wiley and Sons. HER2, human epidermal growth factor receptor 2. EGFR, epidermal growth factor receptor. Negative antibody: reproduced with permission from Chu et al. (2019), Copyright 2019, Royal Society of Chemistry. Peptides: reproduced with permission from Peng et al. (2017), Copyright 2017, American Chemical Society. MNPs, magnetic nanoparticles: reproduced with permission from Tian et al. (2019), Copyright 2019, American Chemical Society. DOPA, 3,4-dihydroxy-L-phenylalanine, a key functional amino acid in mussel adhesive proteins: reproduced with permission from Zhong et al. (2021), Copyright 2021, American Chemical Society. LAPTM4B, lysosomal protein transmembrane 4 β with extraordinarily high expression level in a majority of solid tumors. AP2H, a LAPTM4B-targeting peptide. Aptamers: reproduced with permission from Chen et al. (2019), Copyright 2019, American Chemical Society; reproduced with permission from Dharmasiri et al. (2009), Copyright 2009, John Wiley and Sons; reproduced with permission from Qin et al. (2020), Copyright 2020, John Wiley and Sons. TDNs, tetrahedral DNA nanostructures.

Immunoaffinity-based platforms usually use molecular probes such as antibodies (Yin et al., 2018; Chen et al., 2019; Chu et al., 2019; Cui et al., 2019), peptides (Peng et al., 2017; Tian et al., 2019; Zhong et al., 2021), and aptamers (Chen et al., 2019; Dharmasiri et al., 2009; Qin et al., 2020) for CTC enrichment and isolation. A simple summary of the nanomaterial-based platforms for the immunocapture of CTCs is shown in Figure 1.

### 2.1 Antibody-Based Immunocapture Platforms

Immunocapture of CTCs is based on the highly specific interaction between capture ligands and the associated antigens that are specifically expressed on the membranes of CTCs. Compared with normal cells or blood cells, CTCs specifically express EpCAM (Joosse and Pantel, 2013), which is a transmembrane glycoprotein overexpressed in most solid cancers and is usually used as a biotarget in CTC isolation strategies. Positive binding ligands are widely used in immunoaffinity approaches, such as anti-EpCAM–functionalized nanoparticles (Huang et al., 2016; Rao et al., 2018; Meng et al., 2019), nanostructured substrates (Wang et al., 2009; Hsiao et al., 2014), and microfluidic chips (Liu et al., 2015; Yu et al., 2015). These platforms achieve highly specific immunocapture of CTCs from solid cancers, such as liver, breast, pancreatic, stomach, prostate, bladder, and colon cancers (Went et al., 2004). Alternatively, some tissues express their own specific membrane proteins, which can also be used for the specific immunocapture of CTCs. For example, prostate-specific membrane antigen (PSMA), specifically expressed in prostate carcinoma (Maurer et al., 2016), can be used to detect CTCs from prostate cancer.

Although positive identification has been mostly utilized for CTC isolation, these methods still suffer from several limitations. For example, EpCAM expression changes occurring during the epithelial–mesenchymal transition (EMT) process of the metastatic cascade increase the migration and invasion ability of cancer cells, inhibiting EpCAM expression (Gorges et al., 2012), so the affinity, which depends on EpCAM expression, will lead to underestimation of CTCs and potentially miss critical subpopulations (Eslami-S et al., 2020). Moreover, Hyun et al. (2016) have shown that EMT-induced breast cancer cells have smaller masses and sizes but possess increased EMT markers and cancer stem cell markers. These cells may be more resistant to chemotherapeutic agents (Mani et al., 2008), which have a close relationship with cancer metastasis. Therefore, the heterogeneities in CTCs may affect the choice of isolation platforms. For this case, microfluidic chips, such as a parallel multi-orifice flow fractionation chip (Hyun et al., 2016) and the Parsortix™ system (Gorges et al., 2016), have more advantages than anti-EpCAM–based methods to simultaneously isolate EpCAM-positive and EpCAM-negative CTCs based on their physical features.

In addition, negative enrichment, capturing nontarget cells to separate target cells, also has advantages for the isolation of CTCs undergoing EMT. Because the densities of WBCs overlap with those of CTCs, it is extremely difficult to separate WBCs from CTCs during primary centrifugation of whole blood. WBCs are usually identified as target cells in negative enrichment techniques, and CD45 membrane antigens are always utilized for the immunocapture of WBCs. Jung’s group (Hyun et al., 2013) developed a geometrically activated surface interaction (GASI) chip and functionalized the whole surface of the microchannel with biotinylated CD45 antibodies. They used these GASI-negative isolation chips to successfully separate 90.67% of MCF-7 cells and first enriched CTCs from metastatic cancer patients. Chu et al. (Chu et al., 2019) proposed a 3D-printed monolithic device and a commercial membrane filter for the direct negative enrichment of CTCs from whole blood, and the viability of tumor cells from simulated samples reached ∼90%.

Furthermore, combining different markers for CTC isolation is another method to increase the sensitivity of epithelial and mesenchymal CTC detection. Other epithelial markers (HER2, HER3, EGFR, and MUC1) (Chen et al., 2019; Thege et al., 2014; Scharpenseel et al., 2019) and some mesenchymal markers (vimentin and N-cadherin) (Kang et al., 2018; Po et al., 2018) can be used together with EpCAM antigen. Zhao’s group (Huang et al., 2018) reported multifunctional microbead-based anti-EpCAM and anti-CD146 antibodies to enhance the capture of heterogeneous CTCs, and mesenchymal CTCs with low EpCAM expression could be isolated with integrity.

Although antibodies are widely used for the immunocapture of CTCs, highly increasing the sensitivity for CTC detection, most of these platforms still have some disadvantages that cannot be ignored; for example, immunocaptured CTCs are difficult to release with integrity, and the common anti-EpCAM–based platform may miss mesenchymal CTCs and EpCAM-negative CTCs.

### 2.2 Peptide-Based Immunocapture Platforms

Over recent decades, a variety of recognition peptides that play key roles in ligand–receptor and protein–protein interactions have also been utilized in CTC isolation. Compared with traditional antibodies, peptides are smaller, more stable, and easier to synthesize with functionalization in large amounts, so they tend to perform better in CTC detection. Yang’s group (Bai et al., 2014) determined that peptide-based nanomaterials have a capture efficiency (∼90%) and purity (∼93%) comparable to those of anti-EpCAM–based positive platforms. They designed a series of peptides to target EpCAM-overexpressing cancer cell lines, and Pep10 (VRRDAPRFSMQGLDACGGNNCNN) was chosen as a recognition peptide in flow cytometry. After bonding Pep10 to magnetic nanoparticles (MNPs), the capture yield of the Pep10@MNP platform reached approximately 90% by isolating MCF-7, SK-BR-3, PC3, and Hep G2 cells from mimic cancer blood samples. Furthermore, Yang’s group (Peng et al., 2017) also designed recognition peptide H13 (GRQLFDNPDQALLDTANDG) to target human epidermal growth factor receptor 2 (HER2). HER2 is overexpressed in approximately 20–30% of human breast and ovarian cancers and is closely related to cancer metastasis (Riethdorf et al., 2010). By using selected H13@MNPs, the capture efficiencies of SKBR3 and SKOV3 cells from mimic cancer blood samples reached 68.56 and 79.26%, respectively. The results demonstrated the high binding affinity of the recognition peptide and protein.

Although peptides are promising molecules to target cells and can maintain the viability of CTCs, their conformational flexibility and small structures sometimes lead to weak interactions with target cells. Moreover, peptide-based immunoaffinity MNPs still attach to the cell surface after isolation, and this phenomenon has been shown in Bai’s article (Bai et al., 2014), causing cytotoxicity in the subsequent culturing process. How to release isolated cells with viability by using peptide-based immunocapture platforms is worth deep exploration.

### 2.3 Aptamer-Based Immunocapture Platforms

Recently, aptamers, synthetic oligonucleotide ligands or single-stranded DNA/RNA molecules have been screened by using SELEX (systematic evolution of ligands by exponential enrichment) (Tuerk and Gold, 1990), to recognize various targets with high affinity and specificity, such as proteins, tissues, and cells (Ding et al., 2020). Over the past few years, many aptamers specific to biomarkers on the membranes of cancer cells, such as EpCAM (Chen et al., 2019), PSMA (Dharmasiri et al., 2009), and HER2 (Qin et al., 2020) biomarkers, have been designed. Moreover, captured targets can be gently released using nuclease hydrolysis treatment or by conveniently adding the aptamer’s competing complementary sequence. Compared with antibodies, aptamers are cheaper and can be easily synthesized in large quantities and modified with various functional groups.

Guo’s group (Yu et al., 2015) combined aptamer-functionalized MNPs with a microfluidic chip to purify CTCs from the whole blood of cancer patients. The MNPs could be directly detached from the cell surface after exonuclease treatment, the final purity of released cells reached up to 86.6%, and the viability of cells after enzymatic treatment only decreased from 82.5 to 71.4%. Furthermore, after culturing the enriched cells with or without enzymatic treatment, the exonuclease had little influence on cell viability, whereas the membranes of cells attached to MNPs broke after 3 days. CTCs acquire mesenchymal markers and lose epithelial markers in the abovementioned EMT process, therefore, capturing different phenotypes of CTCs simultaneously is a challenge. Pei’s group (Gao et al., 2020) synthesized two kinds of aptamer-modified MNPs, SYL3C-MNPs and NC3S-MNPs, both of which were used to capture epithelial and mesenchymal CTCs. The capture efficiencies of MCF-7 cells and HeLa cells (a high expression of N-cadherin and low expression of EpCAM) reached 92.5 and 92.0% in mimic cancer blood samples, respectively. They successfully isolated CTCs from 15 out of 16 clinical blood samples while using the dual aptamer-based platform, indicating the hopeful application of multifunctional aptamer platforms in clinical detection.

Many aptamer-based platforms have been used to isolate CTCs, but until now, there have been very few useful aptamers, limiting their clinical application. Aptamers are traditionally selected by using purified proteins as target molecules, which may be different from their native forms in tertiary structure, causing the recognition of the same proteins expressed on the surface of target cells, which is an adverse effect (Nutiu and Li, 2003). In future work, more precise aptamers will be selected to further improve the capture efficiency of live cells as in cell-SELEX (Gao et al., 2020), which uses whole cells as selection targets to achieve the high affinity and specificity of generated aptamers. On the other hand, there is an urgent need for stable probes to improve the detection precision of CTCs in whole blood.

## 3 Functional Immunoaffinity Nanomaterials for Circulating Tumor Cell Isolation and Detection

Ongoing development of nanomaterials provides many advantages in enhancing CTC enrichment efficiency and detection sensitivity and includes adding various chemical groups to the surface that are easily modified with multifunctional ligands, and the large surface area of nanomaterials beneficially increases the density of capture agents, which will greatly improve the capture efficiency of target cells.

### 3.1 Nanoparticles

**FIGURE 2:**
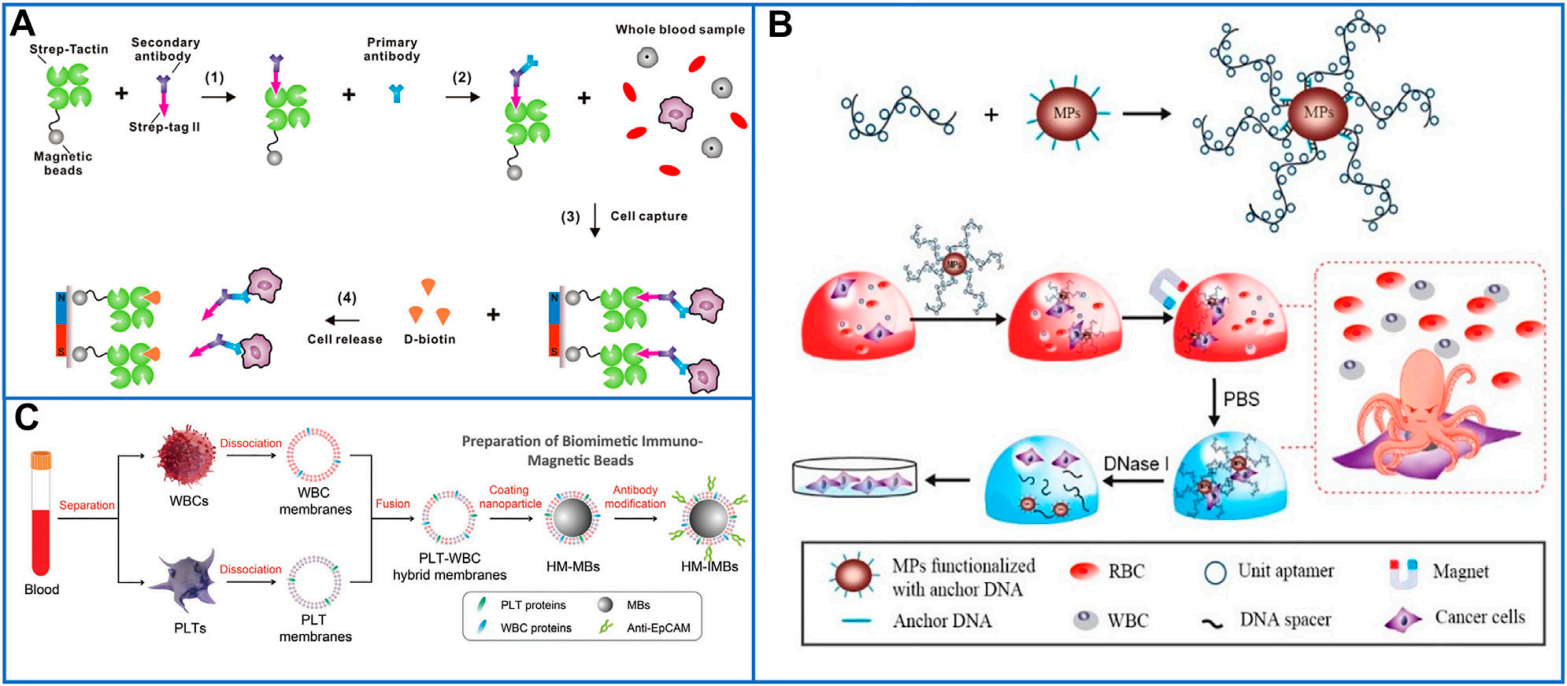
Magnetic nanoparticles to immunocapture CTCs. **(A)** Platform based on biotin-triggered decomposable immunomagnetic beads to efficiently capture and release viable CTCs: reproduced with permission from Lu et al. (2015), Copyright 2015, American Chemical Society. **(B)** A “NanoOctopus” platform based on long multimerized aptamer DNA strands to mimic octopus's tentacles for enhancing the sensitivity and specificity of immunomagnetic beads: reproduced with permission from Chen et al. (2019), Copyright 2019, American Chemical Society. **(C)** Schematic of PLT and WBC–hybrid membranes–modified immunomagnetic beads for highly enhancing the purity of the captured CTCs. Reproduced with permission from Rao et al. (2018), Copyright 2018, John Wiley and Sons.

To date, numerous magnetic platforms have been successfully developed to detect DNA/mRNA (Perez et al., 2002; Grimm et al., 2004), proteins (Perez et al., 2002), drugs (Tsourkas et al., 2004), and tumor cells (Lee et al., 2009) with perfect sensitivity. Even though commercial kits based on magnetic activated cell sorting (MACS^®^) microbeads have been developed for CTC enrichment and detection from whole blood (Königsberg et al., 2011), magnetic nanoparticles (MNPs) have also attracted great attention for CTC enrichment because their high surface-area-to-volume ratio will provide more bonding sites for captured agents, which may further enhance their capture yield (Cardoso et al., 2018). As discussed before, the release of captured CTCs without damage is important for *ex vivo* culture and may offer an opportunity for personalized cancer therapy. Huang’s group (Lu et al., 2015) introduced biotin-triggered decomposable immunomagnetic beads to make the release of viable CTCs possible. After preparing Strep-Tactin (a mutated streptavidin molecule) conjugated to magnetic beads (STMBs), chemically synthesized Strep-tag II (a short peptide sequence) was oriented and conjugated with anti-EpCAM antigens that specifically interacted with STMBs to capture CTCs. To release cells with high viability, D-biotin was added to break the interaction between Strep-tag II and STMBs because D-biotin has a higher affinity for Strep-Tactin than Strep-tag II. This capture and release system is shown in Figure 2A.

Chen et al. (2019) developed a “NanoOctopus” platform to enhance the sensitivity and specificity of immunomagnetic beads by using long multimerized aptamer DNA strands to mimic octopus tentacles. Each DNA tentacle had hundreds to thousands of repeating aptamer units spaced by 20T sequences, and these spacers decreased the frequency of aptamer misfolding, ensuring a high capture efficiency without steric hindrance. A schematic of this platform is shown in Figure 2B. Utilizing this system, the cell capture efficiencies reached ∼95 and 88% ± 6% in PBS buffer and mimic clinical samples, respectively. After DNase treatment for 20 min, 87.7 ± 6% of the captured cells had been released, and 94% of the recovered cells remained viable after 7 days of culture. The platform has potential for commercialization; MNPs and biotinylated DNAs are commercially available, and their isolation method is also quick, high-throughput, and cost-effective.

Biocompatible and stable core-shell nanoparticles, such as MnO_2_ (Xiao et al., 2017), metal-organic frameworks (Xie et al., 2019), and hydrogels (Wang et al., 2021), have also been developed to simply capture and release viable cells through magnetic fields, and could be chosen as shells to coat on MNPs. Zhao’s group (Xiao et al., 2017) reported an effective approach by coating an MnO_2_ layer film on MNPs (MNPs@MnO_2_), and the anti-EpCAM could be efficiently conjugated on the core-shell nanoparticles mainly through functional hydroxy groups on the surface of MnO_2_. Moreover, the MnO_2_ layer could be easily dissolved by extremely low concentrations of oxalic acid at room temperature without damaging the captured cells, which successfully realized the separation of viable cells from the MNPs. However, there were still many background WBCs attached to the inorganic layers because of nonspecific adsorption, thus resulting in the low purity of the target cells. Recently, Pei’s group (Wang et al., 2021) proposed antifouling hydrogel-coated MNPs to isolate CTCs from clinical blood samples with high purity and viability. In this platform, MNPs were first linked with 3-(trimethoxysilyl)propyl methacrylate. Then, a hydrogel film (zwitterionic sulfobetaine methacrylate) was directly synthesized on the surface of MNPs to inhibit the adhesion of nontarget cells, and methacrylic acid was chosen as the active film to be coated on the MNPs to provide carboxyl groups, which could be functionalized with anti-EpCAM through NH_2_-S-S-biotin. After glutathione (GSH) solution treatment, disulfide bonds were broken to release cells with good viability, and more than 96% of the recovered cells maintained viability in the study of mimic clinical blood samples.

According to the wide utilization of biomimetic core-shell nanoparticles in a variety of biomedical applications, another antifouling platform of blood cell membrane–coated magnetic beads (MBs, ∼100 nm) was well designed to isolate CTCs with highly improved purity due to the biologically repulsive interaction between WBCs and blood cell membranes (Rao et al., 2018; Meng et al., 2019). Rao et al. first used platelet and WBC hybrid membranes (HM) to fabricate HM-MBs, which could be modified with biotinylated anti-EpCAM through 1,2-distearoylsn-glycero-3-phosphoethanolamine-N-[methoxy(polyethylene glycol)-2000]-COOH. The preparation process of biomimetic and immunomagnetic beads (HM-IMBs) is shown in Figure 2C (Rao et al., 2018). In cell line isolation studies, the capture efficiency of these core-shell nanoparticles was 95%, which is much higher than the 66.5% capture efficiency of commercial Dynabeads IMBs. While isolating CTCs from peripheral blood samples of cancer patients, the HM-IMB platform also exhibited higher efficiency and purity than Dynabeads IMBs. However, this HM-IMB platform is unable to release viable CTCs, which makes *ex vivo* culturing more difficult.

**FIGURE 3:**
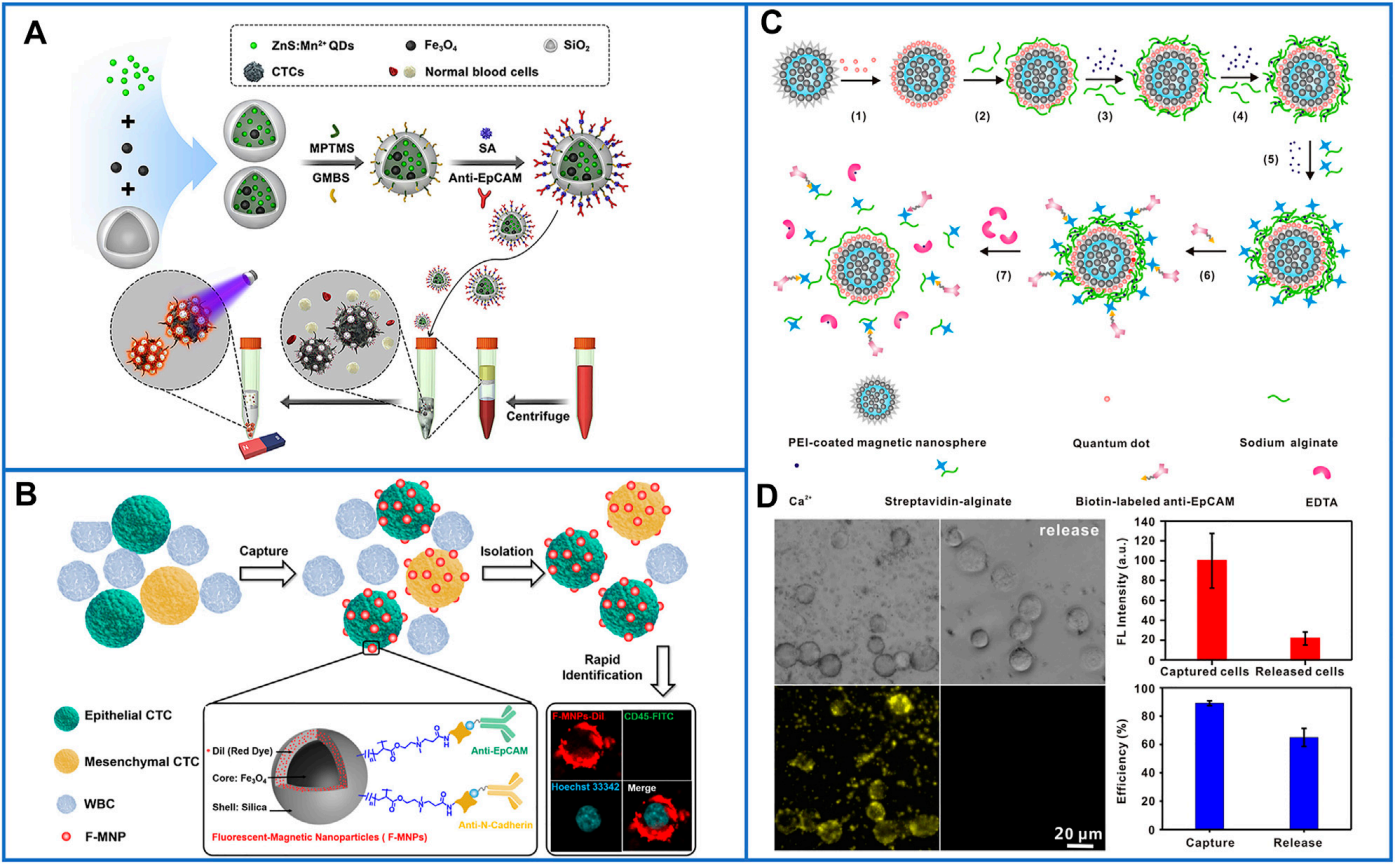
Platforms based on skillfully designed fluorescent-magnetic nanoparticles for isolating and identifying CTCs. **(A)** Equipping ZnS:Mn^2+^ quantum dots and magnetic nanoparticles into hollow SiO_2_ nanospheres for capturing and conveniently identifying CTCs. Reproduced with permission from Cui et al. (2019), Copyright 2019, Elsevier. **(B)** Schematic of Fe_3_O_4_@SiO_2_ core-shell nanoparticles decorated with DiI dyes, and dual-antibody (anti-EpCAM and anti-N-cadherin) used for identifying CTCs from whole blood samples in only one-step processing. Reproduced with permission from Wang et al. (2019), Copyright 2019, American Chemical Society. **(C)** Schematic of fluorescent-MNPs coated by Ca^2+^-initiated alginate for capturing and identifying CTCs, and releasing viable CTCs. Reproduced with permission from Xie et al. (2014), Copyright 2014, American Chemical Society. **(D)** Microscopic images of isolated cancer cells and fluorescent identification by using Ca^2+^-initiated alginate-based fluorescent-MNPs, the two figures on the right of **(D)** show the capture yield and release yield of this platform. Reproduced with permission from Xie et al. (2014), Copyright 2014, American Chemical Society.

In the above studies, the methods for the detection and enumeration of CTCs usually rely on traditional three-color immunocytochemistry (ICC) identification, which leads to disruption of the viability and biological functions of captured CTCs. In addition, the additional step of staining cells may cause the loss of target cells, especially for patient blood samples, and the loss of CTCs may seriously influence the detected results. Recently, fluorescent-magnetic nanoparticles were skillfully designed to isolate and identify CTCs with high efficiency (Cui et al., 2019; Xie et al., 2014; Wang et al., 2019). Cui et al. (2019) introduced ZnS:Mn^2+^ quantum dots (ZnS:Mn^2+^ QDs) and MNPs into hollow SiO_2_ nanospheres. The captured cells were conveniently identified by strong orange fluorescence due to ZnS:Mn^2+^ QDs, and a schematic illustration is shown in Figure 3A. They successfully achieved a capture efficiency of 90.8% in mimic cancer blood samples and detected 5–29 CTCs/mL in nine clinical blood samples. To further improve the capture efficiency, Wang et al. (2019) used a dual-antibody (anti-EpCAM and anti-N-cadherin) interface to target epithelial CTCs and mesenchymal CTCs. The fluorescent platform, as shown in Figure 3B, was composed of Fe_3_O_4_@SiO_2_ core-shell nanoparticles and DiI dyes (red fluorescence) decorated on SiO_2_ shells and could also identify CTCs from whole blood samples in a one-step process. In mimic blood samples, the capture efficiency was approximately 98.8%, while 3–27 CTCs/ml were detected in clinical blood samples, and the purity of the captured CTCs reached 0.2–6%, which made them feasible for subsequent molecular analysis. To release cells from nanoparticles with viability, Huang’s group (Xie et al., 2014) first presented a new strategy using engineered nanobioprobes. In their work, Ca^2+^-initiated layer-by-layer self-assembly was employed to deposit alginate coatings on fluorescent-MNPs, which can be easily decomposed by EDTA treatment. The modified steps are shown in Figure 3C. Microscopic images of cancer cells isolated and detected by this fluorescent platform are shown in Figure 3D. No fluorescence was observed after EDTA treatment, demonstrating the disconnection between antibodies and fluorescent MNPs.

Even though MNPs have been widely used for the simple isolation of CTCs in magnetic fields, other nanoparticles have also been designed for the high-purity capture and release of CTCs. The density gradient centrifugation method based on gelatin nanoparticle-coated silicon microbeads (SiO_2_@Gel MBs) was skillfully developed by Huang et al. (2018). Gelatin nanoparticles have been proven to be easily functionalized with antibodies (such as anti-EpCAM) through their carboxy groups, and gelatin can be mildly degraded by matrix metalloproteinase-9 enzyme (MMP-9) treatment for the release of captured cells with integrity (Huang et al., 2016). Unlike MNPs, silicon microbeads are larger and have a higher density for easy separation from CTCs, releasing CTCs without nanoparticles attached for further precise analysis. To enhance the capture of CTCs with extremely low EpCAM expression, Huang et al. (2018) used anti-EpCAM and anti-CD146 antibodies conjugated to SiO_2_@Gel MBs. This novel method showed a capture efficiency of 80 and 85% purity. After MMP-9 treatment, up to 94% of the captured CTCs were released with 92.5% viability. Utilizing this simple method for isolating CTCs from clinical blood samples, one out of 10 colorectal cancer cases and two out of 10 breast cancer cases were positive for the 3140A/G (*H1047R*) heterozygous mutation in the *PIK3CA* oncogene, which demonstrated that this approach had the potential for applications in personalized cancer diagnostics.

### 3.2 Fractal Substrates

Fractal structures are widespread in tissue microenvironments, such as the nanoscale components [microvilli (Wang et al., 2014) and filopodia (Woo et al., 2003)] on the cell surface, especially the filopodia on the surface of the CTCs can provide the capacity for their further deformation, adhesion, and migration (Yim et al., 2005; Mattila and Lappalainen, 2008). Inspired by these natural structures, artificial fractal structures based on nanoscale features have attracted more and more interests for their appealing applications in isolation of CTCs. Because the increased surface area in fractal structures could provide more binding sites for affinity capture, these offer a simple and low-cost solution for enhancing the performance of CTCs enrichment. In order to ensure high CTC capture efficiency, the topographic nanostructures of underlying substrates are recommended to match the structures of cellular surface components, such as filopodia (Mattila and Lappalainen, 2008).

#### 3.2.1 Fractal Substrates Based on Nanoparticles

**FIGURE 4:**
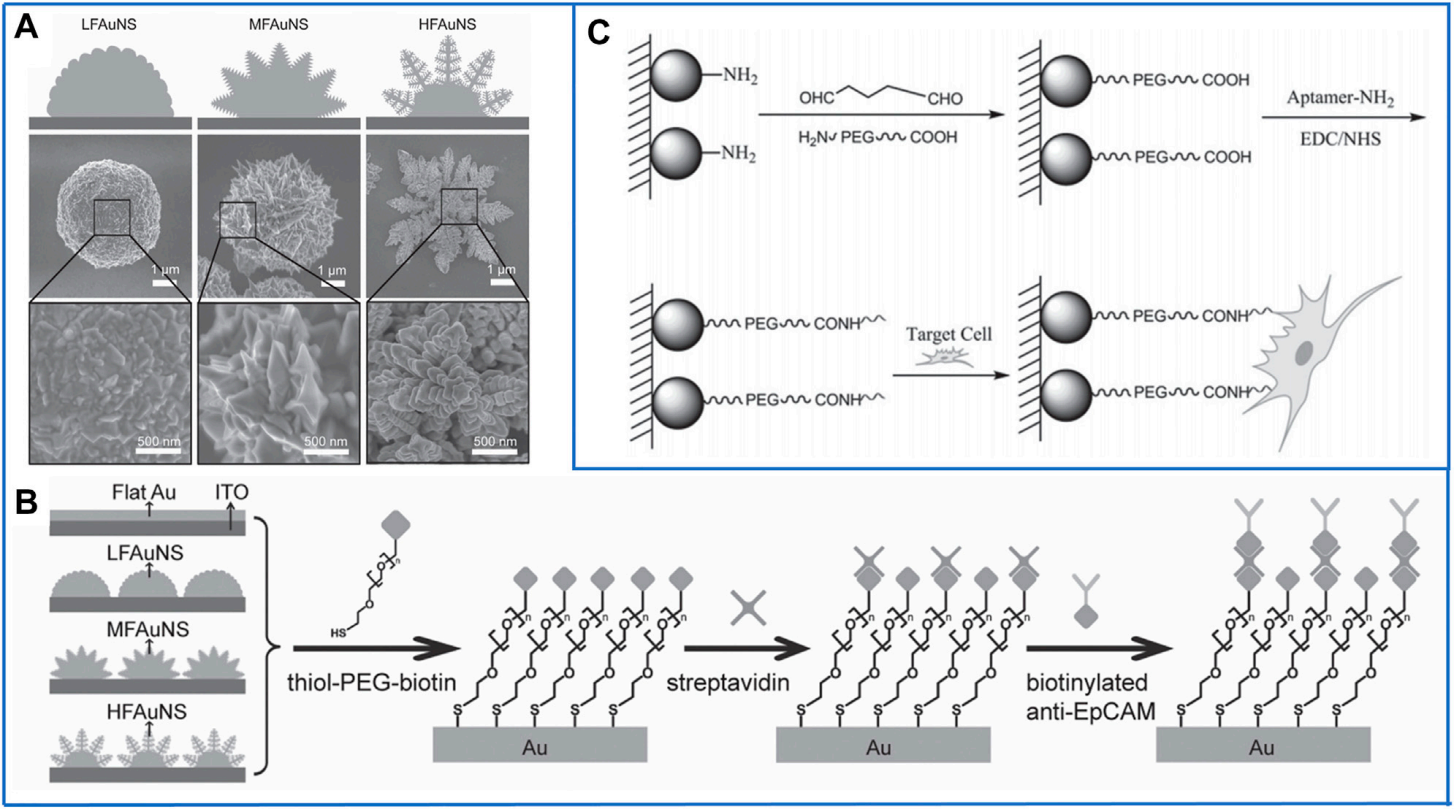
Fractal substrates based on nanoparticles to immunocapture CTCs. **(A)** Scanning electron microscope (SEM) images of three kinds of fractal gold nanostructures (FAuNSs) to capture and electrochemically release CTCs. Reproduced with permission from Zhang et al. (2013), Copyright 2013, John Wiley and Sons. **(B)** Schematic diagram of the chemically modified method on the surface of FAuNSs for capturing CTCs. Reproduced with permission from Zhang et al. (2013), Copyright 2013, John Wiley and Sons. **(C)** Fractal substrates based on chitosan nanoparticles and the chemically modified method for capturing CTCs. Reproduced with permission from Sun et al. (2015), Copyright 2015, John Wiley and Sons.

In 2013, Wang’s group (Zhang et al., 2013) presented a kind of fractal gold nanostructure (FAuNS) to mimic nanoscale filopodia on the surface of cancer cells, which showed outstanding recognition of CTCs from whole blood. Three kinds of FAuNSs were generated by simply adjusting potentials in a one-step electrochemical deposition approach (Figure 4A). Compared with a flat Au substrate, FAuNSs with large fractal structures showed a capture efficiency of 62 ± 13%, which was as much as 21 times that of the flat Au substrate. To release the captured cells with good viability, thiol-poly(ethylene glycol)-biotin molecules were employed to strongly combine with Au because they can not only be tightly conjugated to streptavidin for further linking with biotinylated antibodies (Figure 4B) but also cleave the sulfur–gold bonds through a simple electrochemical process. In studies testing the HFAuNS-based cell release system on EpCAM-positive MCF-7 cells, 98% of the captured cells were released after a potential of −1.2 V was applied for only 5 min, and 95% of the released cells remained viable.

Soon after, in 2014, Zhao’s group (Cheng et al., 2014) designed transparent and excellent biological nanoparticles on a traditional glass substrate, and the transparent property of the captured substrate made it possible to directly observe the growth behavior of target cells under optical microscopes. The biological nanoparticles were composed of hydroxyapatite and chitosan (HA/CTS), which are both widely used in bioengineering applications, such as tissue engineering (Menon and Mukherjee, 1995) and antimicrobial applications (Sharma et al., 2020). After conjugation with biotinylated anti-EpCAM, the capture efficiency of EpCAM-positive HCT116 cells was as high as 88%. In studies of artificial blood samples, the captured cells adhered to HA/CTS began to proliferate and migrate after culturing for 4 days, and after 14 days, the captured cells proliferated significantly. Furthermore, 11 of 12 clinical peripheral blood samples were detected as CTCs by using this platform. Unfortunately, the captured cells could not be released for downstream cancer diagnosis, so at the same time, other researchers in Zhao’s group (Huang et al., 2014) designed transparent substrates based on MnO_2_ nanoparticles to realize the release of the captured cells. MnO_2_ nanoparticles with a diameter of 200 nm were self-assembled on a glass substrate with a thin monolayer film, which not only exhibited a high degree of transparency but also improved the affinity between the cells and substrate. Moreover, this monolayer could be easily reduced by oxalic acid due to its 150-nm thickness. The capture efficiency and release efficiency of this platform reached 80.9% and 92 ± 2%, respectively. The viability of the released cells could reach up to 90%, which demonstrated that the inorganic nanosubstrates had potential application in the isolation of rare cells with great integrity.

Despite the above inorganic nanomaterials, organic nanomaterials may be more biocompatible for isolating cells to retain their maximum viability. In 2015, Pei’s group (Sun et al., 2015) first fabricated chitosan nanoparticles on a transparent substrate by using electrospray technology, which is simple for producing a large area. Moreover, a bifunctional polyethylene glycol (PEG) was linked onto the amino groups of chitosan nanoparticles, which not only played a role as an “antifouling” molecule for decreasing nontarget cell adhesion but also introduced carboxyl (-COOH) on the surface of chitosan nanoparticles. Similar to MNPs, -COOH could be easily activated by EDC/NHS for immobilizing DNA aptamers, and the schematics are shown in Figure 4C. Even though 90% of EpCAM-positive MCF-7 cells could be captured and 95% of the captured cells remained viable, it is impossible to release them from this platform. In the next few years, Pei’s group also developed some nanostructured substrates based on organic nanoparticles, such as folic acid-modified polystyrene nanospheres (Chen et al., 2019) and hydrogel nanoparticles (Wang et al., 2021). All of these platforms achieved high capture efficiency and successfully realized *in situ* culture of the captured cells, but it is difficult to release the captured cells without damage because the molecular chains of organic nanoparticles are much longer; harmful organic solvents must be used to degrade them. Thus, it is valuable for some researchers to study how to release cells without damage due to the outstanding advantages of organic materials.

#### 3.2.2 Fractal Substrates Based on Nanopillars/Nanowires/Nanorods

**FIGURE 5:**
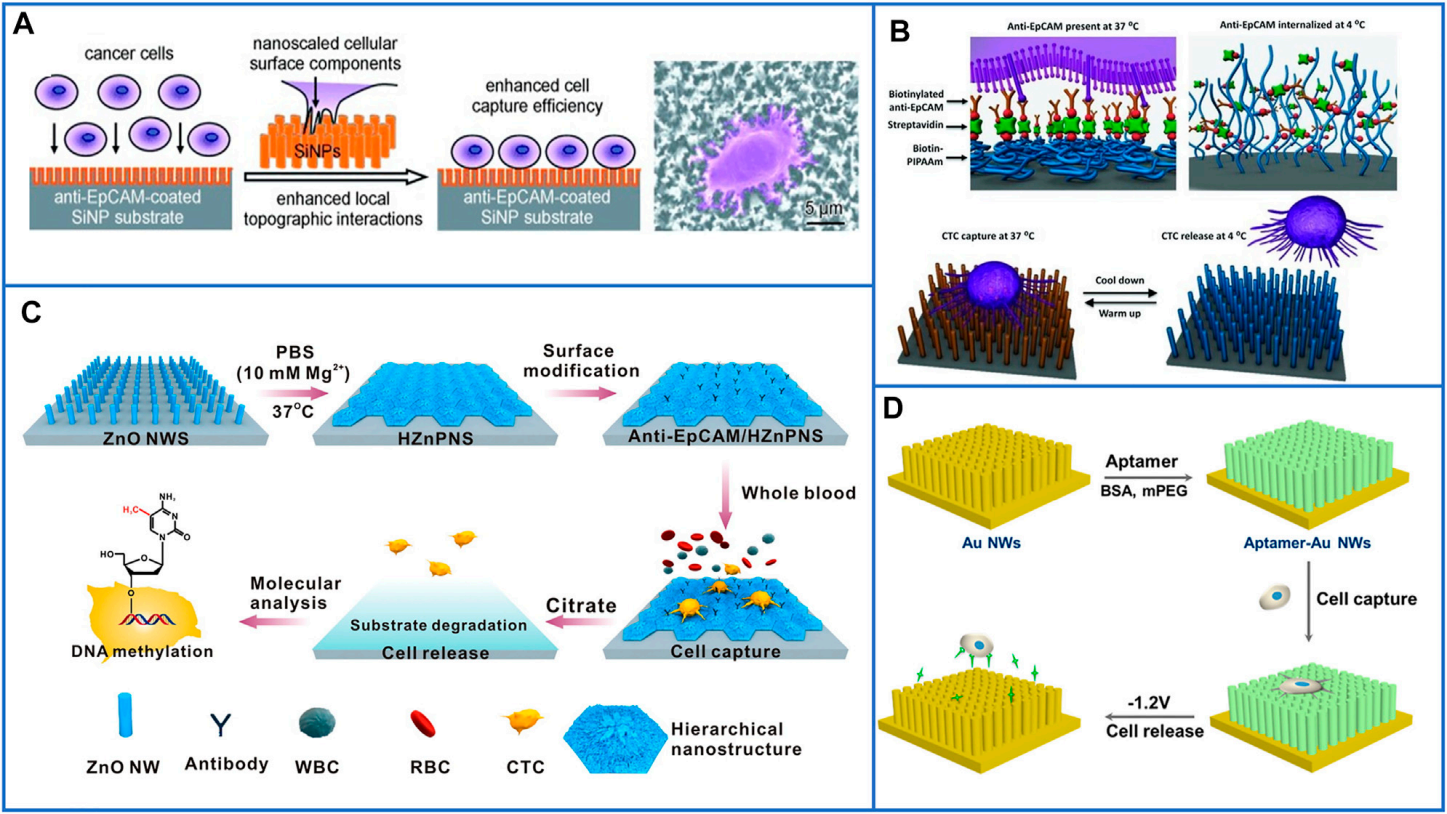
Fractal substrates based on nanopillars and nanowires to immunocapture CTCs. **(A)** The platform based on silicon-nanopillars array for highly enhancing the capture yield of CTCs. Reproduced with permission from Wang et al. (2014), Copyright 2009, John Wiley and Sons. **(B)** Schematic of silicon nanowire substrate coated with thermally responsive PIPAAm for capturing and releasing CTCs with high viability. Reproduced with permission from Hou et al. (2013), Copyright 2013, John Wiley and Sons. **(C)** A flowerlike substrate based on ZnO NWs coated with Mg^2+^ solutions to capture, rapidly release, and molecularly analyze viable CTCs. Reproduced with permission from Guo et al. (2016), Copyright 2016, American Chemical Society. **(D)** A platform based on AuNWs for capturing and releasing viable CTCs by using gently electrochemical method. Reproduced with permission from Zhai et al. (2017), Copyright 2017, American Chemical Society.

A platform based on a vertically oriented silicon-nanopillar (SiNP) array was first demonstrated by Tseng’s group in 2009 (Wang et al., 2009). The SiNPs were fabricated by chemical etching technology, and their diameters were 100–200 nm, which allowed for enhanced local interactions between cells and the nanostructured Si substrates; the illustration is shown in Figure 5A. After conjugation with anti-EpCAM to capture cells, many interdigitated cellular protrusions of the captured cells could be clearly observed on the SiNP substrate, while the cells on the flat Si substrate almost appeared round in shape. As a result, the capture efficiency of the SiNP substrate was ten times that of the flat Si substrate, which suggested that nanostructured substrates could possibly be used for enhancing cell capture yields. To study the CTC-derived molecular signatures and functional analysis of CTCs, Tseng’s group (Hou et al., 2013) in 2013 developed a new platform based on a silicon nanowire substrate (SiNWS) and thermally responsive polymer brushes for realizing the release of immobilized cells from capture agent-coated substrates. Poly(N-isopropylacrylamide) (PIPAAm), a thermally responsive polymer, was polymerized *in situ* on SiNWSs by using monomer solution, and then, biotin was conjugated to PIPAAm to tightly link streptavidin. Similarly, biotinylated anti-EpCAM was introduced onto the functional SiNWS through a biotin–streptavidin interaction, as illustrated in Figure 5B. At 37°C, the cells adhered to the hydrophobic domains of PIPAAm, and then, the substrate was cooled to below 4°C. The backbone of PIPAAm would extend and become hydrophilic toward the solute, realizing the release of captured cells. By using this platform, more than 90% of EpCAM-positive cancer cells could be captured, and approximately 90% of the released cells remained viable.

Inspired by the enhanced communication between cells and bioelectronic interfaces, Hsiao et al. (2014) developed a platform based on conductive micro/nanorod arrays for capturing cancer cells. By using a vertical silicon nanowire (SiNW) substrate as a template, tosylate-doped poly(3,4-ethylenedioxythiophene) (PEDOT:TOS) could be easily fabricated with micro/nanostructures through a poly(dimethylsiloxane) (PDMS) transfer printing technique, and rods of different sizes were fabricated to explore the different affinities between cells and substrates. As a result, the cell-capture efficiency increased with decreasing rod diameter from the micro- to nanoscale, which demonstrated that the nanostructured substrates exhibited better affinity to cancer cells. However, these above nanofabrication methods are complex and usually require elaborate designs, and Huang’s group (Guo et al., 2016) designed a degradable zinc-phosphate-based hierarchical nanosubstrate (HZnPNS) to enhance CTC capture performance and make gentle cell release possible. First, zinc oxide nanowires (ZnO NWs) were grown on the surface of a glass substrate by using a simple low-temperature hydrothermal method. Then, ZnO NWs were coated with PBS containing Mg^2+^ to form flowerlike HZnPNSs. After modification with carboxylic groups, biotinylated anti-EpCAM could be conjugated on the hierarchical substrate. The detailed process is shown in Figure 5C. In their study, this hierarchical nanostructured substrate matched better with the filopodia of cancer cells than a single vertical ZnO NW substrate and exhibited significantly enhanced cancer cell capturing efficiency as high as 90 ± 1% through simultaneous immune-affinity and topographical interactions. Moreover, the substrates could be rapidly dissolved by biocompatible sodium citrate to release captured cells with a high viability of 92 ± 1%. Furthermore, downstream molecular analysis of isolated CTCs from 11 breast cancer patients was demonstrated, and the results showed that the contents of 5-methyl-2ʹ-deoxycytidine in CTCs of breast cancer patients are lower than those of healthy controls, which made this HZnPNS platform promising for personal cancer therapy.

In Section 3.2.1, we introduced a substrate based on Au nanoparticles that could enhance the cell-capture yield and release captured cells through an electrochemical process. Similar to this design, Zhai et al. (2017) developed aptamer-modified gold nanowire arrays (AuNWs) to capture and release human leukemic lymphoblasts (CCRF-CEM). The AuNWs were electrochemically deposited on conductive glass by using anodic alumium oxide (AAO) as the template, and the length or diameter of the AuNWs could be easily controlled by changing the deposition parameter or AAO structure. AuNWs exhibited a much higher capture efficiency than a flat Au substrate. Moreover, the Au-S bonds could be easily broken through electrochemical reduction desorption, as shown in Figure 5D. As a result, 96.2% of captured cells were quickly released in −1.2 V for 30 s, and the cells maintained a high viability of 90%. However, the isolation method based on gold substrates is certainly expensive. Thus, the properties of low cost and simple operation need to be further considered in designing CTC-isolated platforms.

**FIGURE 6:**
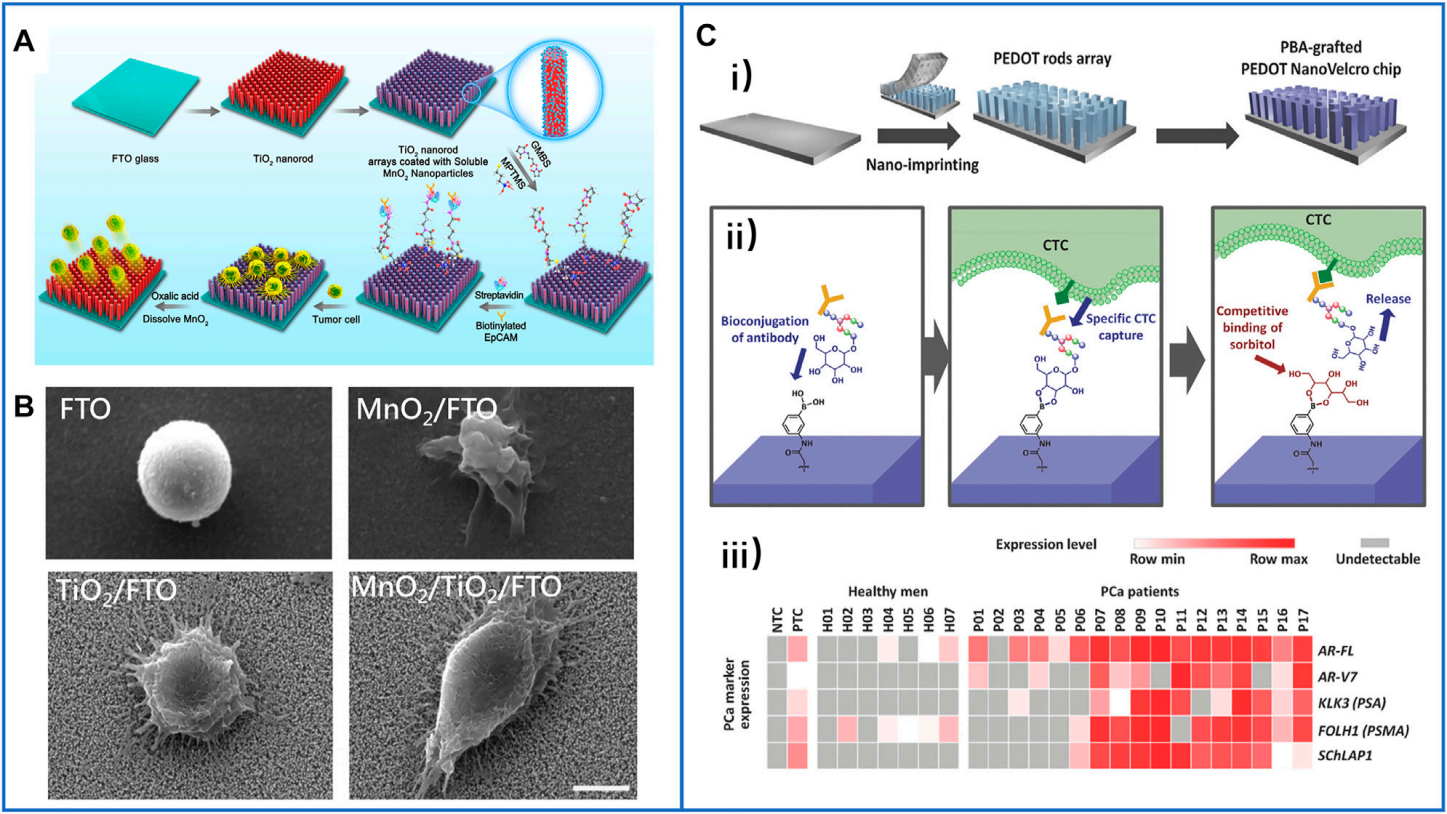
Fractal substrates based on nanorods to immunocapture CTCs. **(A)** The schematic process of TiO_2_ nanorod arrays coated with degradable MnO_2_ nanoparticles and conjugated with antibodies to capture and release CTCs. Reproduced with permission from Li et al. (2018), Copyright 2018, American Chemical Society. **(B)** SEM images of captured cancer cells on different substrates of FTO, MnO_2_/FTO, TiO_2_/FTO, and MnO_2_/TiO_2_/FTO. Scale bar: 5 μm. Reproduced with permission from Li et al. (2018), Copyright 2018, American Chemical Society. **(C-i)** Schematic illustration of PBA-grafted PEDOT NanoVelcro chip; **(C-ii)** the mechanism of conjugating antibody on PBA for CTCs capture and release; **(C-iii)** summary of RNA signature detection in blood samples of seven healthy men and CTCs of 17 PCa patients. Reproduced with permission from Shen et al. (2018), Copyright 2018, John Wiley and Sons.

In 2018, Li et al. skillfully coated MnO_2_ nanoparticles (MnO_2_ NPs) on TiO_2_ nanorod arrays (Li et al., 2018), and the principle of this platform is clearly shown in Figure 6A. MnO_2_ NPs could not only be conjugated with anti-EpCAM to capture target cells but also be dissolved by using oxalic acid to release cells. Moreover, the TiO_2_ nanorod arrays synthesized by the hydrothermal method greatly enhanced the physical affinity between the cells and the nanosubstrate, as shown in Figure 6B. Through this inexpensive platform, the cell-capture yield reached 92.9%, and the release efficiency reached 89.9%. Furthermore, the cell-isolated performance of the MnO_2_/TiO_2_ nanorod substrate was nearly the same as that of immune magnetic beads in isolating CTCs from the clinical blood of breast cancer patients, which demonstrated the great potential of this platform in the detection of CTCs and further cancer diagnosis.

Unlike TiO_2_ nanorod arrays, ZnO nanowire arrays could be dissolved with a mild sodium citrate solution treatment that we have introduced before. Therefore, directly conjugating antibodies on ZnO nanowire arrays to capture cells without an additional coating layer is simpler, but the release of viable cells also needs to be proven. In 2020, Cui et al. designed a PDMS substrate with micro gear pillar structures using soft lithography, on which they grew vertical ZnO nanowire arrays to isolate cells (Cui et al., 2020). It was obvious that the 3D platform had a larger surface area to provide antibody binding sites which could highly enhance the capture efficiency. However, in the process of releasing captured cells, PDMS was hardly degraded, even though ZnO had been dissolved completely. As a result, some cells may have remained in the microgrooves of the PDMS gear, decreasing the release efficiency, and only 90% of the captured cells could be successfully released, which is lower than that of other nanosubstrate-based platforms. Inspired by this 3D hierarchical substrate, whole degraded 3D substrates are worth skillfully designing for the capture and release of CTCs.

Furthermore, on-chip purification of CTCs from patient blood and further biomarker detection of isolated CTCs were explored for simple downstream analysis and potential cancer diagnosis. Recently, many studies have demonstrated that DNA mutations can be detected in the analysis of CTCs, such as epidermal growth factor receptor (EGFR) (Sundaresan et al., 2016). However, there is a relatively low abundance of DNA mutations in some kinds of tumors, especially in prostate cancer (PCa) (Robinson et al., 2015). Thus, the detection of gene expression levels and RNA biomarkers has been explored. However, due to the absence of CTCs and interference of many WBCs, obtaining high-quality signals of special biomarkers has become a challenge. In 2018, Shen et al. (Shen et al., 2018) cooperated with Tseng’s group to solve this problem; they fabricated a nanomaterial platform based on a phenylboronic acid (PBA)–grafted PEDOT nanorod array. In which PBA could conjugate antibodies through oligosaccharide residues, while PEDOT applied a 3D nanostructure to highly enhance antibody conjugation to efficiently capture cells. Moreover, the competitive binding of sorbitol-PBA could easily disturb the oligosaccharide-PBA bond to release viable cells, as illustrated in Figures 6C-i,ii). By using this platform, the researchers purified CTCs from the blood samples of PCa patients, and the expression levels of several PCa-specific RNA biomarkers were further analyzed, including *AR-FL*, *AR-V7*, *KLK3*(PSA), *FOLH1* (PSMA), and *AChLAP1*. Almost all of them were expressed at higher levels in the CTC samples of PCa patients, especially in the blood samples of metastatic patients, and the results are shown in Figure 6C-iii). This capacity provides an important foundation for “liquid biopsy” in the clinic.

#### 3.2.3 Fractal Substrates Based on Nanofibers

**FIGURE 7:**
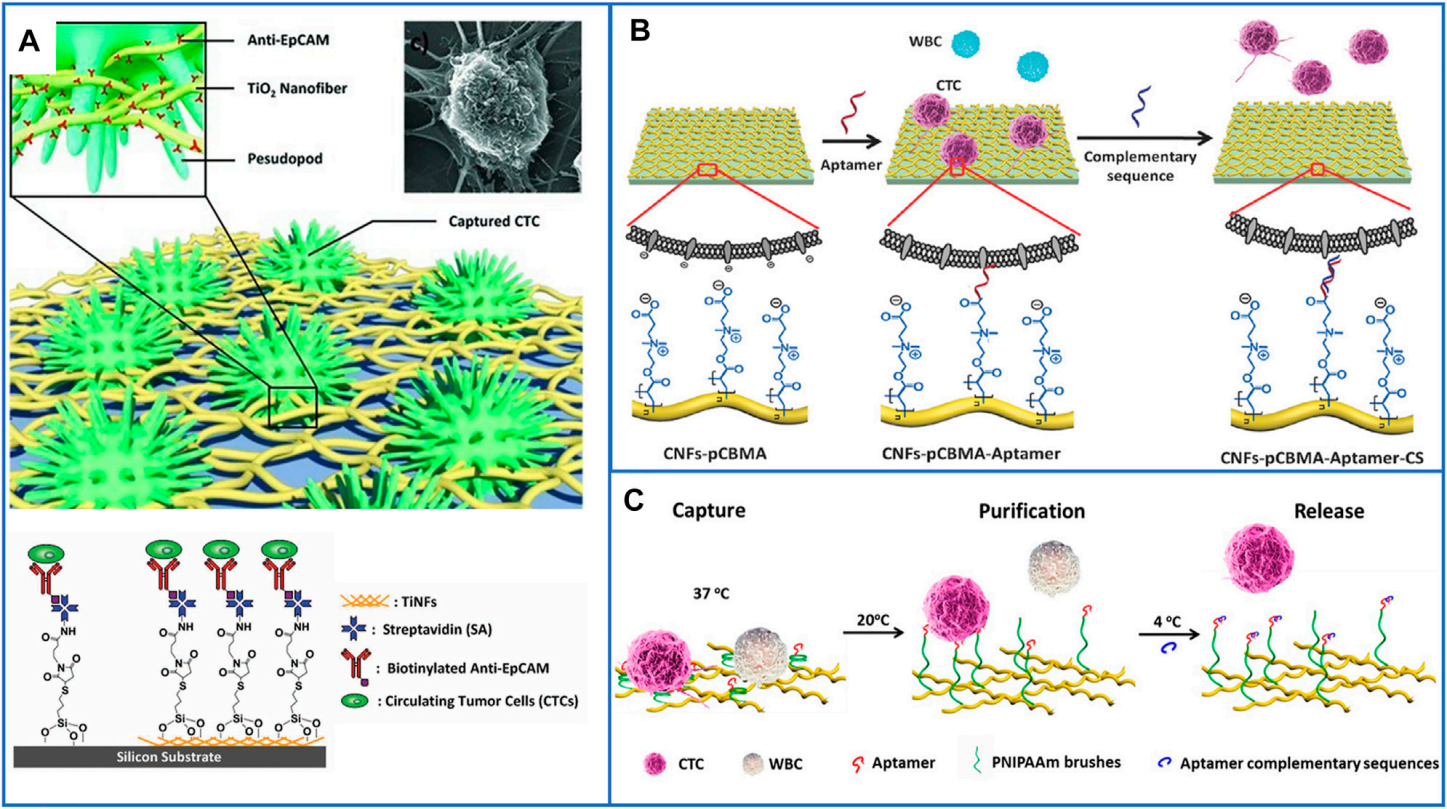
Fractal substrates based on nanofibers to immunocapture CTCs. **(A)** The platform based on horizontal TiO_2_ electrospun nanofibers for highly enhancing capture yield of CTCs. Reproduced with permission (Zhang et al., 2012). Copyright 2012, John Wiley and Sons. **(B)** Schematic illustration of the design functional bio-interface based on chitosan nanofibers grafted with pCBMA brushes for highly enhancing the purity of captured CTCs and releasing cells. Reproduced with permission (Sun et al., 2016). Copyright 2016, John Wiley and Sons. **(C)** Schematic illustration of chitosan nanofibers coated with PNIPAAm for CTCs capture, purification and release. Reproduced with permission (Wang et al., 2017). Copyright 2017, American Chemical Society.

Inspired by the nanoscale features grown on cellular surfaces, such as microvilli and filopodia, nanofibers were skillfully fabricated to mimic these structures to enhance the interactions between live cells and substrates. Zhao’s group (Zhang et al., 2012) first used horizontal TiO_2_ electrospun nanofibers (TiNFs) and biotinylated anti-EpCAM to capture CTCs, which are different from the vertically oriented nanopillars/nanowires/nanorods. The nanofibers better mimicked the function of extracellular matrices, as shown in Figure 7A, and the nanofibers could be deposited onto any substrate. Compared with that of the flat substrate, the capture density of the TiNF-based substrate was enhanced approximately 18 times, and the cells captured from the artificial blood samples enabled more than 45% recovery. To achieve a high purity of isolated cells, Pei’s group (Liu et al., 2020) then modified TiO_2_ nanofibers with an an anti-adhesion molecule (bovine serum albumin, BSA). After conjugating with the nucleolin aptamer AS1411, this platform had a capture efficiency of 83.08 ± 3.84% and purity of 87.77 ± 0.78% for the capture of MCF-7 cells from the artificial blood samples. However, the TiO_2_ NF-based platform could not realize the release of cells.

In 2016, Pei’s group (Sun et al., 2016) fabricated a functional biointerface based on chitosan nanofibers grafted with poly(carboxybetaine methacrylate) (pCBMA) brushes for highly efficient CTC capture and simultaneously decreased nonspecific cell adhesion, as shown in Figure 7B. The capture yields reached approximately 96% with considerable purity (0.3% of WBCs), and 98.1% of the cells could be nondestructively released by introducing a complementary sequence at 4 °C within 50 min. After live/dead staining, the viability of the released cells was approximately 90.5%, which was slightly different from that of the control cells. The next year, Pei’s group (Wang et al., 2017) also introduced thermoresponsive poly(N-isopropylacrylamide) (PNIPAAm) brushes on the surface of chitosan nanofibers to achieve captured cells with high purity and realize the release of cells. It is well known that the structure of PNIPAAm undergoes a sharp transition from compact globules to expanded coils as the environmental temperature decreases from 37 °C to its lower critical solution temperature, so this is an ideal method for preventing nonspecific cell adhesion, as shown in Figure 7C. Furthermore, dual antibodies (anti-EpCAM and anti-N-cadherin) have also been used to modify nanofibers to enhance the capture performance of epithelial and mesenchymal CTCs (Liu et al., 2019), which is the same as the above methods using nanoparticle-based platforms.

#### 3.2.4 Biomimetic Substrates

Many nanomaterial-modified substrates for CTC isolation have been introduced above; even though the capture efficiency has been increased by nanostructured materials, the interactions between the nontarget blood cells and nanomaterials lead to nonspecific adhesion, which will seriously decrease the purity of the captured CTCs. Thus, the subsequent detection or characterization of CTCs is affected in precise clinical diagnosis. Multiple antifouling substrates have been introduced above to restrain nonspecific adhesion of WBCs, but they are almost all synthetic materials. Recently, Pei’s group (Ding et al., 2020) used three types of cancer cell membranes to develop a naturally biomimetic substrate to capture target CTCs. The capture efficiency of this platform reached 90%, and the purity of the isolated cells reached up to 97% when mixing CTCs and WBCs at a ratio of 1:1, which provided an interesting strategy using a natural interface for the isolation and release of CTCs with high purity.

## 4 Microfluidic Chips Based on Immunoaffinity Nanomaterials for Circulating Tumor Cell Isolation

With the development of a simple but powerful microfluidic handling system, microfluidic technologies have emerged as an active field for isolating CTCs with high purity. Due to the constant flow of microfluids, nonspecific cells are effectively removed during the process of capturing cancer cells (Cheng et al., 2019), as in CTC-chips (Nagrath et al., 2007), herringbone-chips (Stott et al., 2010), vortex chips (Sollier et al., 2014), NanoVelcro (Dong et al., 2020), and so on, which have been skillfully designed to isolate and detect CTCs. Nanomaterials used as functional components can also be flexibly integrated in the microchannel to provide more bonding sites for conjugation with cancer-cell capture agents to enhance the capture efficiency during CTC isolation.

**FIGURE 8:**
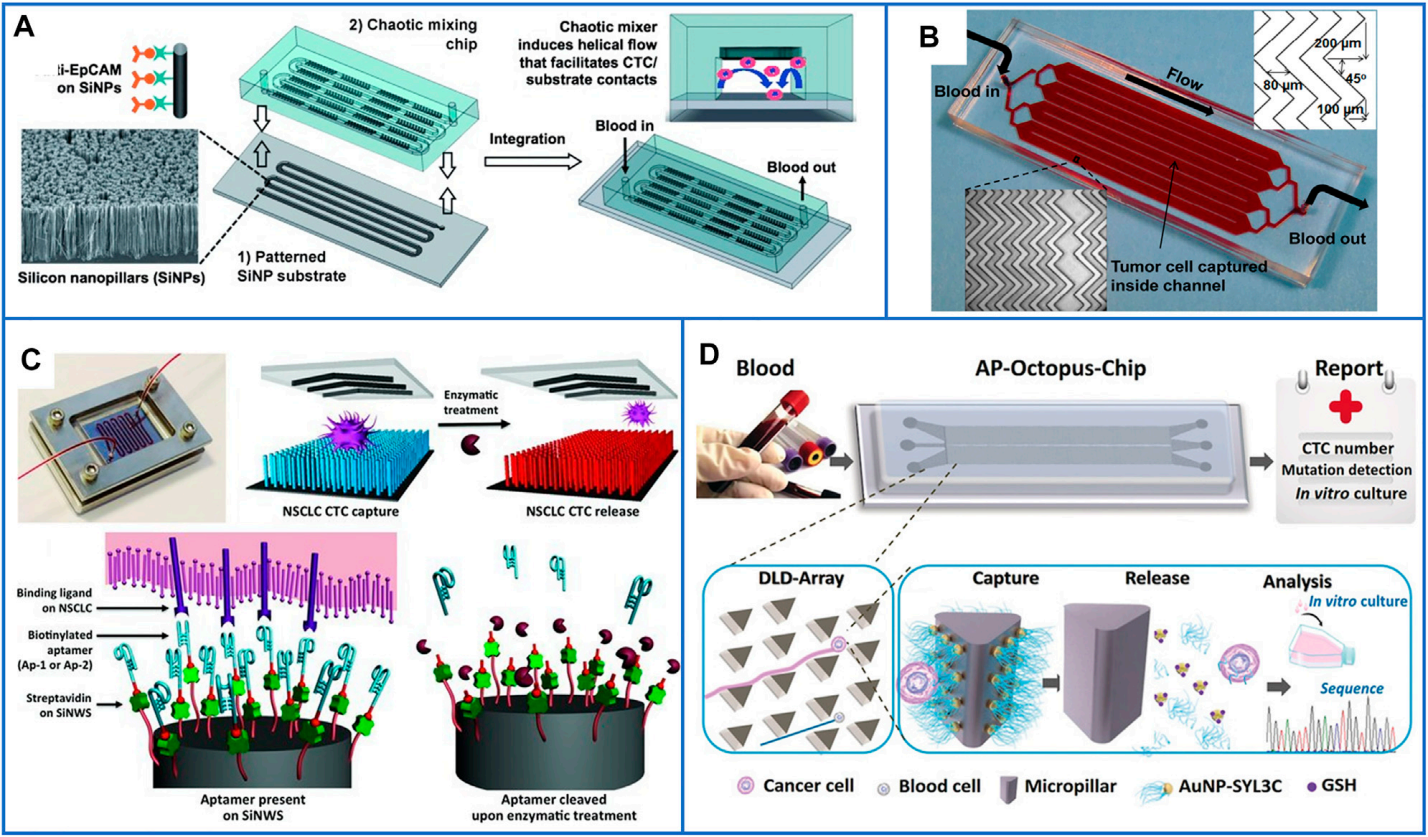
Microfluidic chips based on immunoaffinity nanomaterials for CTC isolation. **(A)** Schematic illustration of a microfluidic chip based on SiNP substrate and PDMS serpentine chaotic microchannel for capturing CTCs with high purity. Reproduced with permission from Wang et al. (2011), Copyright 2011, John Wiley and Sons. **(B)** A photo of microfluidic chip with herringbone mixers and AuNPs, the dimensions are shown in the top, right corner of the figure. Reproduced with permission from Sheng et al. (2013), Copyright 2013, American Chemical Society. **(C)** A microfluidic chip based on PDMS chaotic mixer and SiNWs substrate conjugated with two biotinylated DNA-aptamers for capturing and releasing CTCs. Reproduced with permission from Shen et al. (2013), Copyright 2013, John Wiley and Sons. **(D)** Working principle of the AP-Octopus-Chip based on AuNP-SYL3C–modified micropillar. Reproduced with permission from Song et al. (2019), Copyright 2019, John Wiley and Sons.

In 2011, Tseng’s group (Wang et al., 2011) first fabricated a patterned SiNP substrate and overlaid a PDMS chip with a serpentine chaotic microchannel to increase the contact frequency between antibodies and cancer cells; the illustration is shown in Figure 8A. This platform had an excellent capture efficiency of 95% and captured more CTCs in patient blood samples than the CellSearch^®^ assay. Since then, many nanomaterials with different structures and functions have been integrated into microfluidic chips to effectively enhance CTC capture efficiency and purity. For example, biocompatible TiO_2_ nanoparticles were used to increase the surface roughness of microfluidic channels and bond with anti-EpCAM antibodies; more than 80% of cells were isolated, but only 50% of them were alive (He et al., 2013). Sheng et al. (2013) also demonstrated that gold nanoparticles (AuNPs) had an increase of 39-fold in binding with DNA aptamers compared with flat microchannels, and they also added herringbone structures to microchannels to increase the contact frequency between aptamers and cells, the platform is shown in Figure 8B. The capture efficiency of this platform reached 92%, and it enabled a high capture efficiency of 93% and purity of 70% from whole blood, even at a high flow rate. Moreover, graphite oxide–coated MNPs loaded in a Ni micropillar-based microfluidic chip have also been developed to capture cells with high efficiency (Yu et al., 2013). Moreover, Xu et al. (2017) first used a microfluidic chip embedded with hyaluronic acid (HA)–functionalized PLGA nanofibers to capture HeLa cells (EpCAM-negative cells) with high efficiency, and the captured HeLa cells could be cultured in the nanofiber-based chip for several days with viability, which may expand the frontiers of functional nanomaterials in diagnostic applications.

To further release CTCs from microfluidic chips, many researchers have first chosen aptamer-based capture agents, which can be digested by a genetically engineered endonuclease, to acquire viable CTCs (Shen et al., 2013; Zhao et al., 2013; Yu et al., 2015; Song et al., 2019; Wu et al., 2021). Shen et al. (2013) used a SiNW-based substrate and a PDMS-based chaotic mixer to form a “NanoVelcro” chip to improve local interactions and the contact frequency between flow-through CTCs and the substrate. Two biotinylated DNA aptamers selected by A549 cells were employed as capture agents to sensitively capture CTCs, and the nanosubstrate-immobilized CTCs could be released by treating with a nuclease solution. A schematic is shown in Figure 8C. Second, nanomaterials that can be gently degraded, such as MnO_2_ nanofibers (Liu et al., 2015) and gelatin nanoparticles (Wei et al., 2019), have been grown in the channels of microfluidic chips for the release of viable CTCs. Third, negative-selection–based isolation of CTCs can also be used in microfluidic chips (Chu et al., 2020).

Because of the dynamic capture of cells in microfluidic chips, several cells might wash away before they contact the capture agents, especially at a high flow rate, which seriously decreases the capture efficiency. Thus, nanomaterials and microscale patterned geometries can both be integrated to further enhance the capture performance of microfluidics. Recently, Yang’s group (Song et al., 2019) used AuNPs modified with EpCAM aptamers (SYL3C) as a nanometer-sized functional interface and used a patterned PDMS micropillar array as a micrometer-sized screen to enhance the capture efficiency and reduce the adsorption of nontargeted cells. This microfluidic chip was named AP-Octopus-Chip, and its working schematic principle is shown in Figure 8D. The capture efficiency of this platform reached up to 89.4%. After GSH treatment, the Au-S bond on the surface of micropillars was easily disrupted so that the release of CTCs reached 80% with 96% viability. In addition to PDMS-based microfluidic chips, cheaper poly(methyl methacrylate)–based microfluidic chips also have the potential for the capture and recovery of CTCs from whole blood samples (Yu et al., 2019), in which nanomaterials can still be grown in the microchannels to improve the isolation performance.

**FIGURE 9:**
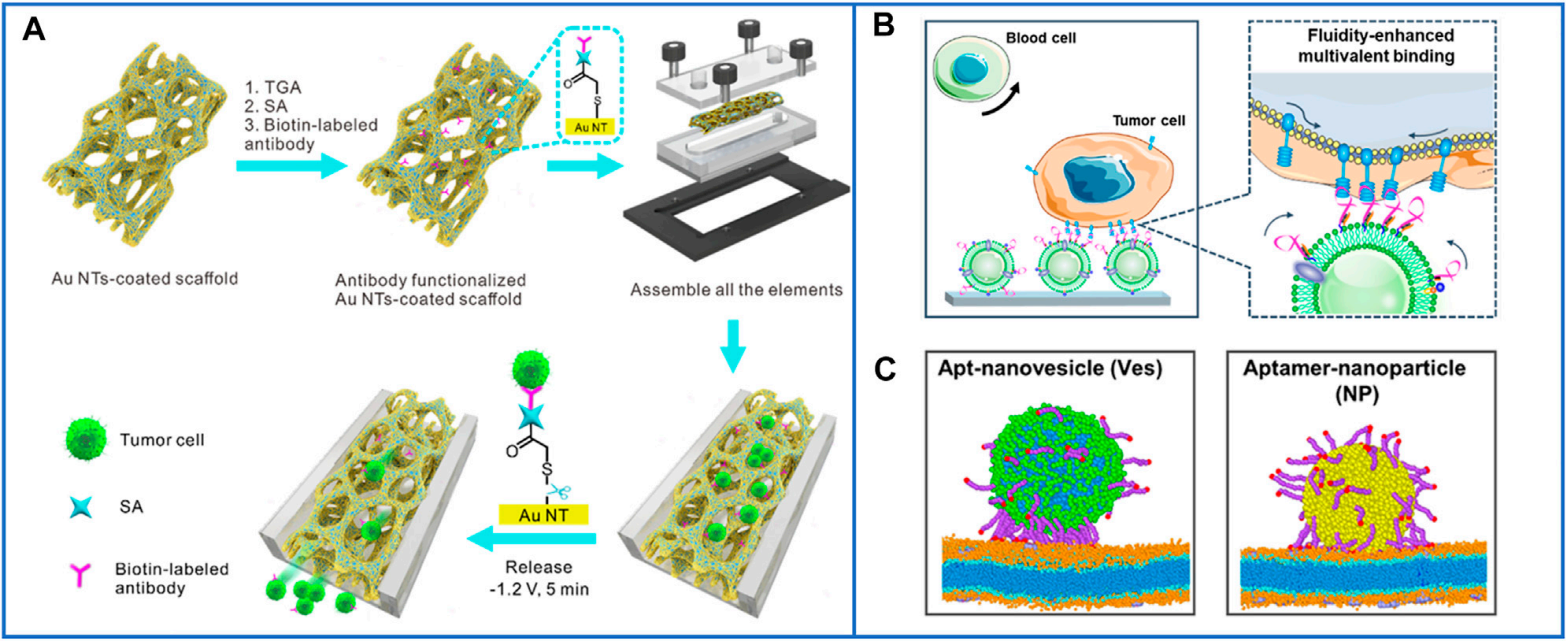
The microfluidic chips based on nanomaterials for highly enhancing the immunocapture efficiency of CTCs. **(A)** A 3D conductive scaffold microchip based on macroporous PDMS and immobilized gold nanotubes for effective capture and recovery of CTCs with high purity. Reproduced with permission from Cheng et al. (2021), Copyright 2021, American Chemical Society. **(B)** Working principle and diagram of the microfluidic chip based on natural nanovesicles for enhancing multivalent binding with cells. Reproduced with permission from Wu et al. (2021), Copyright 2020, American Chemical Society. **(C)** The simulations of the difference of aptamer scaffolds interacting with cell membranes and nanoparticles. Reproduced with permission from Wu et al. (2021), Copyright 2020, American Chemical Society.

To our surprise, Huang’s group (Cheng et al., 2021) used 3D macroporous PDMS as channels and immobilized gold nanotubes (AuNTs) on the surface of PDMS for the capture and release of CTCs. A schematic is shown in Figure 9A. The macroporous structure could change the fluids from laminar to chaotic flow, which could easily improve the contact frequency between capture agents and CTCs, while AuNTs could be linked on the PDMS through Au-S bonds that are usually broken by low-voltage exposure for the release of cells with great purity. By using this platform, the 3D macroporous chip could acquire various types of CTCs, such as individual CTCs, CTC clusters, and CTC-WBC clusters, which might promote a more precise downstream analysis of cancer.

Nanomaterials can supply more bonding sites for capture agents, but the recognition ligand density on the capture interface also directly influences the efficiency of CTC capture. A low ligand density limits the binding affinity, while an excessive ligand density leads to entanglement between ligands and reduces their recognition ability. Recently, Yang’s group (Wu et al., 2020) developed a new microfluidic chip based on natural nanovesicles to capture CTCs, as shown in Figure 9B. Because of the nature of the fluidity of the nanovesicles, the free movement of aptamers on the nanovesicle membrane improved their lateral rearrangement and localization so that the local interfacial concentration of aptamers on the nanovesicles could be regulated dynamically when binding with one or multiple receptors on the cell membrane, as shown in Figure 9C. The aptamers could diffuse from the upper surface to the binding region due to the fluidity of the nanovesicles. Meanwhile, the biomimetic nanovesicles inherited the blood cell resistance capability of the leukocyte membrane, which can decrease their nonspecific adsorption to blood cells and reduce cell damage, facilitating precise downstream analysis.

The detection and characterization of CTCs in whole blood is critical for the analysis of potential biomarkers in cancer diagnosis, and it is challenging to acquire CTCs with great purity and retain high-quality biomarkers. In 2017, Kelley’s group (Poudineh et al., 2017) reported a microfluidic chip based on magnetic ranking cytometry to profile the heterogeneous phenotypes of CTCs from whole blood samples without any pretreatment, and the characterization was performed based on their surface expression phenotype. Similar to the aim of characterizing the subpopulations of CTCs, a new aptamer-mediated capture and antisense-triggered release platform that realized two-dimensional (2D) isolation of CTCs was also reported by Kelley’s group (Labib et al., 2016). In addition to using ICC technology to identify CTCs, Raman imaging technology is also an effective method to characterize cancer cells. Cho et al. (2018) utilized AuNPs conjugated with Raman-active nanoprobes to identify CTCs or circulating cancer stem cells based on their surface marker expression phenotypes. Surface-enhanced Raman scattering (SERS) technology can also be combined with chip-based immunomagnetic isolation for the detection of CTCs. Wilson et al. (2020) used four-color SERS nanotags with different Raman reporters to recognize four different cancer biomarkers of individual tumor cells in whole blood, demonstrating that this platform has the potential for the detection of multiple surface biomarkers on CTCs. Furthermore, to detect the different contents of CTCs, Tseng’s group first used “click chip” systems (Dong et al., 2019) for CTC purification on a chip, and then, reverse transcription (RT) droplet digital polymerase chain reaction (PCR) was performed off the chip. Based on this technology, *ALK/ROS1* and EGFR *T790M* (Wang et al., 2020) mRNA can be detected to guide treatment intervention and monitor the progression of non–small-cell lung cancer (NSCLC).

In addition to the potential commercialization of microfluidic chips, fluidics control and large-scale imaging are more difficult, and CTCs captured inside chambers or channels are difficult to remove. Therefore, these disadvantages might limit their applicability to routine clinical practice. Thus, further developing multifunctional sections on one chip, such as sections simultaneously integrating the isolation, characterization, and analysis of CTCs, may be a solution.

The performance of the above nanomaterial-based immunocapture platforms for CTC isolation is summarized in Table 1.

**TABLE 1:** Summary of nanomaterial-based immunocapture platforms for CTCs isolation.

Category	Materials	Capture agents	Cell lines	Cell line isolation study			Clinical blood sample		Ref.
				Yield	Purity	Viability	Ratio	CTC count	
Nanoparticles	MNPs	Anti-EpCAM	A431	C: 70–86%	84 ± 3%	85%	100%	2–215	Lu et al. (2015)
			SK-BR-3	R: 70%			(*n* = 17)		
	MNPs	EpCAM aptamer	CCRF-CEM	C: 95%	∼97%	94%	100%	-	Chen et al. (2019)
				R: ∼88%			(*n* = 33)		
	MNPs@	Anti-EpCAM	HCT116	C: 83%	-	70%	-	-	Xiao et al. (2017)
	MnO_2_			R: 57%					
	MNPs@	Anti-EpCAM	MCF-7	C: 95%	∼97%	-	95%	5–16	Rao et al. (2018)
	PLT and WBC		HCT116	R: -			(*n* = 20)		
	MNPs@	Anti-EpCAM	MCF-7	C: 96%	-	95%	100%	1–12	Wang et al. (2021)
	Hydrogel			R: 96%			(*n* = 5)		
	ZnS:Mn^2+^ QDs and Fe_3_O_4_/SiO_2_	Anti-EpCAM	MCF-7	C: 90%	-	-	100%	5–29	Cui et al. (2019)
			SW480	R: -			(*n* = 9)		
	Fe_3_O_4_@	Anti-EpCAM and anti–N-Cadherin	MCF-7	C: ∼99%	-	-	100%	3–27	Wang et al. (2019)
	SiO_2_-DiI			R: -			(*n* = 10)		
	SiO_2_@	Anti-EpCAM and anti-CD146	MCF-7	C: 80%	＞85%	∼93%	95%	1–16	Huang et al. (2018)
	Gel MBs		HCT116	R: 94%			(*n* = 20)		
Nanoparticles-based substrates	AuNPs	Anti-EpCAM	MCF-7	C: 62%	-	95%	-	-	Zhang et al. (2013)
				R: 98%					
	HA/CTS NPs	Anti-EpCAM	HCT116	C: 85%	-	-	92%	1–13	Cheng et al. (2014)
				R: -			(*n* = 12)		
	MnO_2_ NPs	Anti-EpCAM	MCF-7	C: 80%	98%	90%	-	-	Huang et al. (2014)
			HCT116	R: 92%					
			A549						
	Chitosan NPs	EpCAM aptamer	MCF-7	C: 90%	∼97%	95%	-	-	Sun et al. (2015)
				R: -					
	NH_2_-PEG-FA NPs	Folic acid	Hela	C: 82%	∼98%	-	-	-	Chen et al. (2019)
				R: -					
	Hydrogel NPs	Anti-EpCAM	MCF-7	C: 87%	-	98%	100%	1–32	Wang et al. (2021)
				R: -			(*n* = 5)		
Nanopillars-/nanowires-/nanorods-based substrates	SiNWs@	Anti-EpCAM	MCF-7	C: 90%	-	90%	-	-	Hou et al. (2013)
	PIPAAm		LNCaP	R: 90%					
			PC3						
	HZnPNs	Anti-EpCAM	MCF-7	C: ∼90%	∼63%	∼92%	92.3%	1–75	Guo et al. (2016)
				R: ∼88%			(*n* = 13)		
	MnO_2_/TiO_2_ nanorods	Anti-EpCAM	MCF-7	C: ∼93%	-	95%	100%	14–32	Li et al. (2018)
			SW480	R: ∼90%			(*n* = 9)		
	Gelatin/TiO_2_ nanorods	Anti-EpCAM	MCF-7	C: ∼93%	-	100%	100%	1–13	Li et al. (2019)
			SW480	R: 100%			(*n* = 10)		
			HepG-2						
	PBA-grafted PEDOT	Anti-EpCAM	LNCaP	C: ∼73%	46%	96%	94%	1–7	Shen et al. (2018)
				R: 95%			(*n* = 17)		
	ZnO NWs	Anti-EpCAM	MCF-7	C: ∼91%	-	96%	100%	3–14	Cui et al. (2020)
			SW480	R: 90%			(*n* = 9)		
Nanofibers-based substrates	Chitosan NFs	DNA aptamer	KATO Ⅲ	C: 96%	-	∼91%	-	-	Sun et al. (2016)
				R: ∼98%					
	Chitosan NFs	EpCAM aptamer	MCF-7	C: 94%	∼100%	∼99%	-	-	Wang et al. (2017)
			CCRF-CEM	R: 95%					
	PLGA NFs	Anti-EpCAM and anti–N-Cadherin	MCF-7	C: 75%	87%	-	-	-	Liu et al. (2019)
			GIST882	R: -					
			CCRF-CEM						
Microfluidic chips	TiNPs	Anti-EpCAM	MGC803	C: 80%	-	50%	100%	2–7	He et al. (2013)
			HCT116	R: 90%			(*n* = 7)		
	SiNWs	DNA aptamer	A549	C: 79%	95%	78–83%	-	-	Shen et al. (2013)
				R: 90%					
	AuNPs	EpCAM aptamer	SW480	C: ∼90%	-	96%	100%	4–18	Song et al. (2019)
			LNCaP	R: 80%			(*n* = 7)		
			KATO Ⅲ						
	AuNTs	Anti-EpCAM	MCF-7	C: ∼93%	-	∼91%	100%	7–103	Cheng et al. (2021)
				R: ∼84%			(*n* = 31)		

C, capture efficiency; R, release efficiency.

Some commercial systems based on immunoaffinity available for CTC isolation have been developed or are being developed. The CellSearch^®^ system, the first automated and standardized system for the detection and quantification of CTCs in the peripheral blood, has been cleared by the United States Food and Drug Administration (FDA) for routine clinical use in metastatic breast cancer patients (Riethdorf et al., 2007). Its cell kit contains ferrofluid particles coated with anti-EpCAM antibodies, and 7.5 ml of blood can be reduced to ∼300 μL containing enriched CTCs after automatic immunocapture processing. In addition to the CellSearch^®^ system, many other EpCAM-based methods have been developed for CTC isolation. For example, MagSweeper (Talasaz et al., 2009), an immunomagnetic cell separator, can easily access and purify circulating epithelial cells for downstream biochemical assays and can process 10–100 ml of blood per hour. The GILPUI CellCollector^®^ (Saucedo-Zeni et al., 2012), an *in vivo* device that captures EpCAM-positive CTCs from the circulating peripheral blood by using a functionalized and structured medical Seldinger guidewire for 30 min, has the potential to enrich CTCs *in vivo*. The IsoFlux platform (Harb et al., 2013) combines microfluidic flow control and immunomagnetic capture to enhance CTC isolation, which can enrich CTCs with sufficient quantity and integrity and even ensure complete transfer of CTCs into the molecular assay, successfully tracking the oncogene mutational changes of patients. The microvortex-generating herringbone-chip (Stott et al., 2010) provides an enhanced CTC capture yield by using herringbone structures to passively mix blood cells in the chip, increasing the number of interactions between the target CTCs and the antibody-coated chip surface. However, some platforms still require clinical validation, and to date, none have been cleared by the FDA except the CellSearch^®^ system.

## 5 Single-Cell Isolation

An increasing number of studies have shown that there are numerous phenotypes in a single tumor, and CTCs from a given patient can possess heterogeneous subpopulations, all of which may be related to the development of cancer metastasis (Song et al., 2017; Keller and Pantel, 2019; Yu et al., 2013). For example, Kong et al. (2021) discovered that genomic heterogeneity occurred in CTCs from metastatic tumors, and the same alterations were undetected in the primary tumor, which demonstrated that the alterations were important for researching metastatic phenotypes. Notably, analysis of heterogeneous CTCs is beneficial for evaluating treatment and disease progression and even implementing personalized treatment during anticancer therapy (Yeo et al., 2016). Therefore, traditional sequencing methodologies (when the cellular information is averaged) does not provide complete information about all heterogeneous CTCs. Fortunately, with the development of cell detection technologies, single-cell analysis can be achieved to obtain information on the genomic heterogeneity in CTCs. Recently, many single-CTC isolation methods have been developed, such as droplet-based technology (Brouzes et al., 2009; Kim et al., 2018), flow cytometry-based technology (Davey and Kell, 1996; Yang et al., 2012), optical tweezer-based technology (Arai et al., 2005; Wang et al., 2013), and acoustic tweezer-based technology (Collins et al., 2015; Guo et al., 2015). Considering our topic, we only introduce immunocapture-based platforms for single-CTC isolation.

**FIGURE 10:**
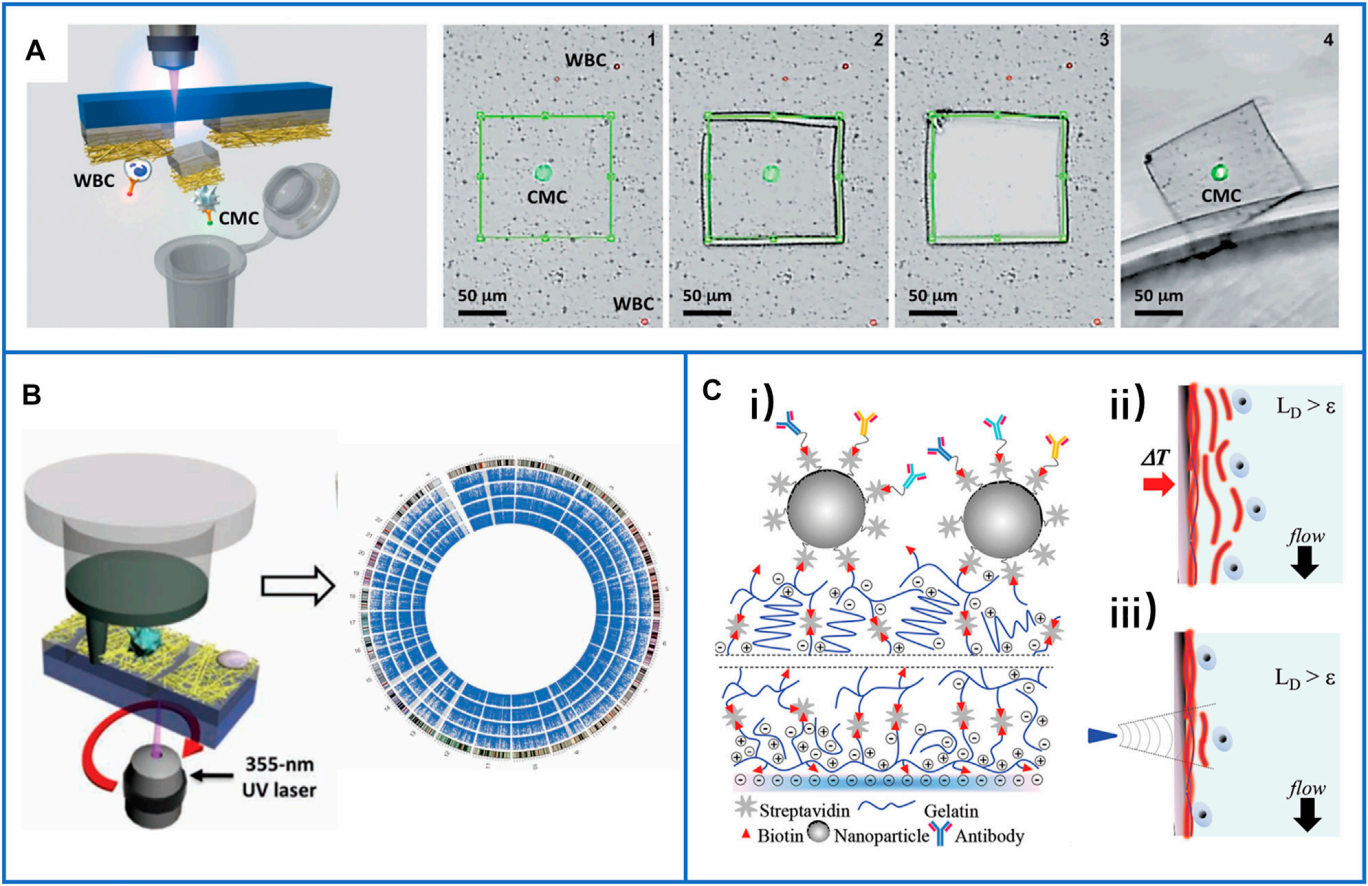
Immunocapture-based platforms for single-cell isolation. **(A)** The process of the NanoVelcro chip with LMD technique to harvest single-CTCs. Reproduced with permission from Hou et al. (2013), Copyright 2013, John Wiley and Sons. **(B)** A polymer nanofiber-embedded microchip with LMD technique to get single-CTCs for the whole exome sequencing to understand the drug-resistant mechanisms of prostate cancer. Reproduced with permission from Zhao et al. (2013), Copyright 2013, John Wiley and Sons. **(C-i)** Schematic of a layer-by-layer nanocoating gelatin-based platform for the immunocapture of CTCs; **(C-ii)** schematic illustration of whole release of CTCs by raising the temperature to 37°C; **(C-iii)** schematic illustration of single-cell release by supplying local mechanical stress. Reproduced with permission from Reategui et al. (2015), Copyright 2015, John Wiley and Sons.

As early as 2013, Tseng’s group introduced the NanoVelcro chip with a highly accurate laser microdissection (LMD) technique to harvest single-CTCs for subsequent Sanger sequence analysis. The substrate target with CTCs could be cut out by using an LMD microscope. The process is shown in Figure 10A (Hou et al., 2013b). They used this platform to acquire single circulating melanoma cells (CMCs) from the peripheral blood samples of two stage IV melanoma patients. The genomic DNA of the CMCs from both patients was amplified, and the results demonstrated that a signature oncogenic *BRAF* ^*V600E*^ mutation was detected in both patients’ single-CMCs, but no *BRAF* ^*V600E*^ mutation was detected in their WBCs, showing the potential of this technique for clinical application. In the same year, Zhao et al. (2013) also used this platform to harvest single-CTCs from PCa patients. After single-CTC isolation, whole-exome sequencing was successfully performed to understand the drug resistance mechanisms (Figure 10B), which showed potential to guide personalized medicine by performing noninvasive liquid biopsies.

In 2015, Reategui et al. presented a layer-by-layer nanocoating gelatin-based platform (Figure 10C-i) for the capture of CTCs and dual-mode release of CTCs (Reategui et al., 2015). Due to the thermosensitivity of gelatin, all captured CTCs could be released by raising the temperature to approximately 37°C (Figure 10C ii), while a microtip could be used to supply local mechanical stress for the release of single-CTCs (Figure 10C-iii). The *PIK3CA* gene of a single breast cancer cell and the *EGFR* gene of a single lung cancer cell were identified, and both had successfully detected mutations.

**FIGURE 11:**
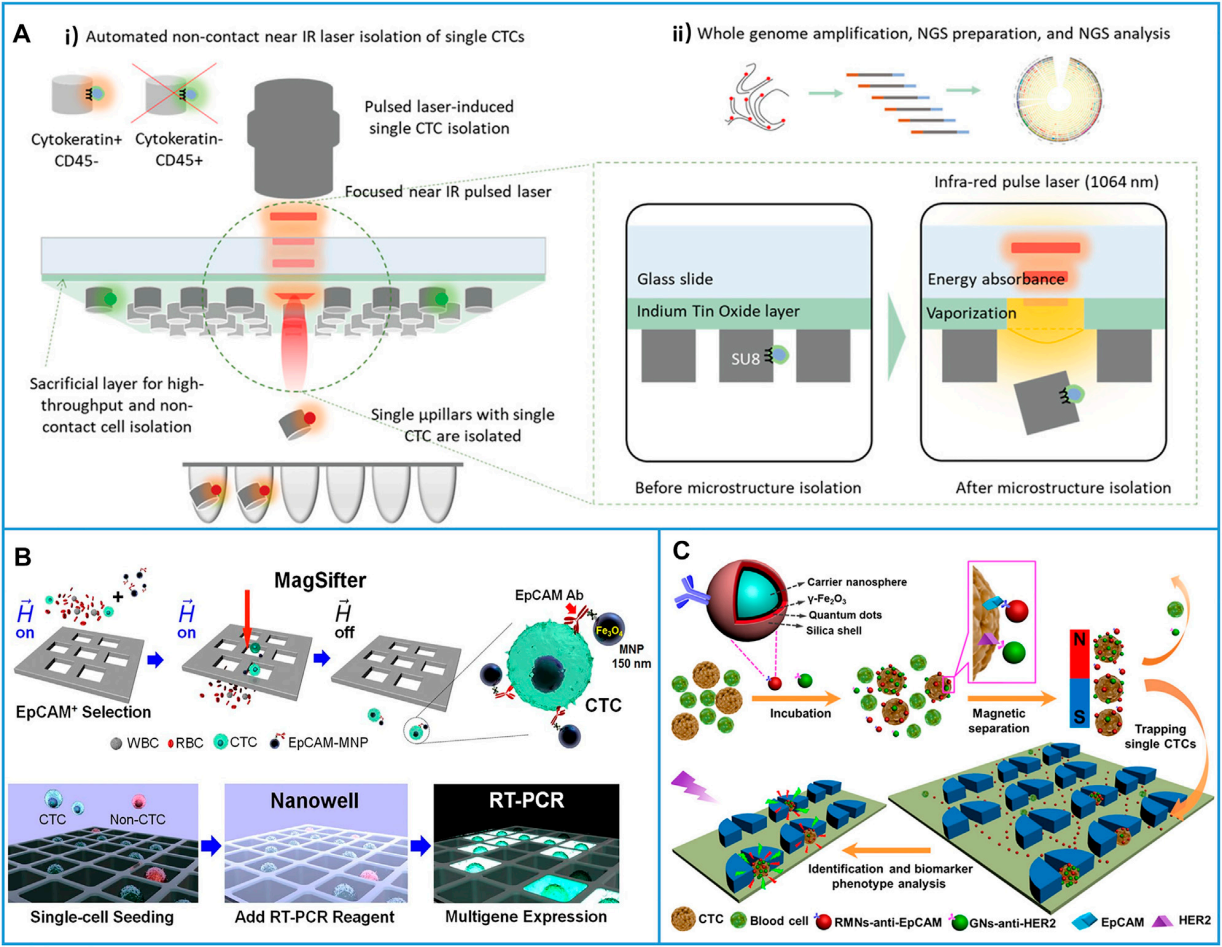
Immunocapture-based platforms for high-throughput single-cell isolation. **(A)** An optomechanically transferrable chip based on a near-IR light beam for single-CTC isolation for whole genome analysis. **(A-i)** Schematic of near-IR isolation of single-CTCs. **(A-ii)** Schematic of the downstream whole genome analysis of a single cell. Reproduced with permission from Kim et al. (2019), Copyright 2019, John Wiley and Sons. **(B)** Platform based on MagSifter and Nanowell devices for high-throughput single-CTC isolation and sensitive detection. Reproduced with permission from Park et al. (2016), Copyright 2016, National Academy of Sciences. **(C)** Micropillars-patterned microfluidic chip for convenient single-cell isolation and rapid *in situ* identification. Reproduced with permission from Wu et al. (2018), Copyright 2018, American Chemical Society.

Due to the manual selection process used for single-CTC isolation, the throughput was seriously limited. Kim et al. (2019) introduced an optomechanically transferrable chip for single-CTC isolation by using a near-infrared (IR) light beam. The CTCs were first immunocaptured in the microchannel, and then, the cells were fluorescently stained with their different proteins to easily identify CTCs from the background cells (Figure 11A-i). Finally, the near-IR beam was used to isolate the target single-CTCs into a PCR tube for downstream whole genome analysis (Figure 11A-ii). Due to the simple process, this platform could be fully automated to realize high throughput (one CTC per second). Through this harmless method, five of 44 isolated single-CTCs had high sequencing results and mutations at the protein level, which demonstrated that this single-CTC platform had the potential for the analysis of heterogeneous CTCs.

On the other hand, combining MNP-based positive CTC isolation with suitable filters is another solution for enhancing single-cell throughput. As shown in Figure 11B, MNPs conjugated with anti-EpCAM were first directly added to the patient blood samples, and then, a MagSifter device was developed to obtain single-CTCs from 2-ml whole blood samples (Park et al., 2016). After seeding the captured cells into a nanowell, single-cell multigene RT-PCR was performed. Through this platform, eight of 11 blood samples from NSCLC patients who spanned all lung cancer stages had positive signals, while ICC only detected three cases in the same samples, which demonstrated that the platform yielded highly sensitive measurements. Considering the advantages of microfluidic chips, Pang’s group (Wu et al., 2018) developed a chip-assisted platform for convenient single-cell isolation and rapid *in situ* identification, as shown in Figure 11C. Here, red fluorescent MNPs conjugated with anti-EpCAM were used to capture CTCs, and green fluorescent nanospheres conjugated with anti-HER2 were used to quantify HER2 expression in different captured cells. After the cells linked with fluorescent MNP fluid through the micropillar patterned microchannel, CTCs could be individually trapped between the micropillars, while other small cells could be washed away, and the difference in the HER2 expression of heterogeneous CTCs could be rapidly identified at the site. This chip-assisted platform provided new opportunities for CTC phenotype analysis.

Single-cell analysis of CTCs is a relatively new method for precise cancer diagnosis and therapy monitoring of cancers, but many factors affect the results of single-cell analysis, such as the isolated performance of platforms. Platforms with a high capture yield and high purity will provide more opportunities for successfully colleting single-CTCs from background blood cells. Moreover, the viability of released cells also affects downstream gene detection. On the other hand, single-CTCs from patient blood samples are difficult to proliferate, which may limit the accuracy of the analysis of their cargoes.

## 6 Conclusion

Due to the rapid development of nanomaterials, many specific and sensitive immunocapture-based platforms have been explored for the effective recognition, isolation, and characterization of CTCs. In this review, we discussed immunoaffinity methods based on nanoparticles, nanopillars, nanowires, nanorods, nanofibers, and 3D micro- and nanostructures linked with different capture agents (antibodies, peptides, and aptamers) for CTC isolation and detection. With the in-depth development of cancer biology and oncology, the inherent heterogeneity of CTCs has been discovered, and efforts in single-cell isolation or on-chip characterization have also been summarized. However, immunocapture approaches for obtaining viable CTCs have some deficiencies. 1) Specific capture agents must be selected for immobilization on particles or substrates, leading to more complex designs compared with physical methods. 2) The methods of releasing CTCs must be skillfully designed to acquire target cells with integrity for reculture or future characterization. The release methods will limit the selection of captured materials. 3) The present immunoaffinity assays are based on epithelial antigens, and their application is limited because cancer cells that lack epithelial antigen expression cannot be detected, and these methods may miss invasive CTCs when cancer cells undergo the EMT process associated with metastasis. Thus, there is an urgent need to explore a better strategy for identifying and isolating tumor cells undergoing the EMT process.

Despite these benefits and deficiencies of nanomaterial-based CTC immunocapture platforms, many challenges prevent clinical transformation. 1) Although many platforms with great isolation performance have been developed, the capture efficiency, viability, purity, and molecular integrity of isolated CTCs are still unmet in terms of the auxiliary diagnosis of cancers. Therefore, many research efforts should be devoted to exploring new nanomaterials, different capture agents, suitable micro- and nanostructures, and better release mechanisms. 2) Along with the in-depth understanding of tumors, new biomarkers on the surfaces of CTCs should be studied, and the circulating extracellular vesicles in the peripheral blood should also be isolated together with CTCs for a more precise analysis of cancers. 3) To deeply study the relationship between the heterogeneous cells of CTCs and the development of tumors, multiple technologies including on-chip characterization or single-cell isolation need to be skillfully designed. 4) Moreover, the lack of standardization in processing samples or analyzing data and the lack of normalization in fabricating nanostructured platforms also lead to serious problems in clinical transformation, such as poor reproducibility of technologies in different research groups. 5) The cost of CTC detection cost may limit its popularization among the general public. However, if CTC assays are reliable enough for the prediction and evaluation of treatment and disease progression, they may reduce other medical costs and improve patient quality of life (Ijzerman et al., 2018). Therefore, it is valuable for different fields of researchers to cooperate together to develop more stable platforms, and academia and industry must both cooperate to efficiently promote standardization and accelerate the transition of immune platforms into clinical applications.

## Data Availability

The original contributions presented in the study are included in the article/Supplementary Material, and further inquiries can be directed to the corresponding author/s.
